# Can the Isothermal Calorimetric Curve Shapes Suggest the Structural Changes in Micellar Aggregates?

**DOI:** 10.3390/ijms21165828

**Published:** 2020-08-13

**Authors:** Katarzyna Łudzik, Sebastian Woloszczuk, Wojciech Zając, Monika Jazdzewska, Andrey Rogachev, Alexander Ivanowicz Kuklin, Anna Zawisza, Małgorzata Jóźwiak

**Affiliations:** 1 Department of Physical Chemistry, University of Lodz, 90-236 Lodz, Poland; malgorzata.jozwiak@chemia.uni.lodz.pl; 2 Frank Laboratory of Neutron Physics, Joint Institute for Nuclear Research, 141980 Dubna, Russia; mojaz@amu.edu.pl (M.J.); rogachev@nf.jinr.ru (A.R.); kuklin@nf.jinr.ru (A.I.K.); 3 Faculty of Physics, Adam Mickiewicz University, 61-614 Poznan, Poland; sebastian.woloszczuk@amu.edu.pl; 4 The Henryk Niewodniczański Institute of Nuclear Physics, Polish Academy of Sciences, 31-342 Cracow, Poland; Wojciech.Zajac@ifj.edu.pl; 5 Moscow Institute of Physics and Technology, 9 Institutskiy per. 141701 Dolgoprudny, Russia; 6 Department of Organic and Applied Chemistry, University of Lodz, 91-403 Lodz Poland; anna.zawisza@chemia.uni.lodz.pl

**Keywords:** SANS, SAXS, ITC curve shape, micellar aggregates, gemini surfactants, structural changes, shape transformation, Monte Caerlo simulation

## Abstract

Inspired by the unusual shapes of the titration curve observed for many surfactants and mixed colloidal systems, we decided to extend the analysis to isothermal titration calorimetric curves (ITC) by paying special attention to potential structural changes in micellar aggregates. In this paper, we used isothermal titration calorimetry in conjunction with Scanning Transmission Electron Microscopy (STEM), Small-Angle Neutron Scattering (SANS) and X-ray Scattering (SAXS) methods support by Monte Carlo and semiempirical quantum chemistry simulations to confirm if the isothermal calorimetric curve shape can reflect micelle transition phenomena. For that purpose, we analysed, from the thermodynamic point of view, a group of cationic gemini surfactants, alkanediyl-α,ω-bis(dimethylalkylammonium) bromides. We proposed the shape of aggregates created by surfactant molecules in aqueous solutions and changes thereof within a wide temperature range. The results provide evidence for the reorganization processes and the relationship (dependence) between the morphology of the created aggregates and the conditions such as temperature, surfactant concentration and spacer chain length which affect the processes.

## 1. Introduction

Isothermal Titration Calorimetry (ITC) has recently become a commonly used technique in the studies of intermolecular interactions essential for cellular activity [1,2,3,4,5]. This sensitive analytical method proved especially useful when monitoring aggregation processes in colloidal solutions [6,7,8,9]. Among amphiphilic compounds, surfactants attract widespread attention for their potential applications in industry and medicine [10,11,12]. The ability of surfactant molecules to self-assemble into micelles in an aqueous environment, capable of taking on a variety of shapes, sizes and maintaining different stability, are among the most useful properties of this group of chemicals. These aggregates (micelles), whose properties are of great importance to fundamental science and the pharmaco-chemical industry alike, stimulate researchers to study their assembly processes as well as the structure and stability [13,14,15]. In this respect, particularly interesting are so-called gemini surfactants (made up of two conventional surfactants joined together by a spacer), whose properties in aqueous and mixed solutions have been investigated since 1990 [16,17,18,19]. Taking into account the reduction efficiency of surface tension, and strong antimicrobial properties [20,21], the group of cationic gemini surfactants, alkanediyl-α,ω-bis(dimethylalkylammonium) bromides, has become especially attractive to scientists. There are many papers devoted to the characteristics of surfactants self-assembly on the basis of their isothermal titration calorimetric results which can bring additional information for the comprehension of their thermodynamic behaviour [22,23,24,25,26]. In 1997, Bijma and Blandamer described three main types of calorimetric titration curves for the demicellisation of aggregate characteristics for different groups of ionic surfactants [26]. One of the most complicated curve shapes is mainly attributed to systems that exhibit non-ideal thermodynamic properties of the solutions. In practice, experiments for surfactants with shorter chains and high value of critical micelle concentration (c.m.c) often yield an unsatisfactory estimation of the c.m.c. Despite an impressive number of papers reporting the use of titration calorimetry to describe the micellisation processes, with enthalpograms analysed within various theoretical frameworks, the origin and interpretation of a specific shape of the calorimetric curve for ionic surfactants are still undeniably open questions [27,28,29]. Fortunately, we presently have the tools at our disposal that enable us to make progress. It has been experimentally proved that aggregates can adopt different geometrical shapes, e.g., spheroids, rods, disks, ellipsoids. The morphology of micelles is determined by the type and structure of the surfactant, solution components, temperature, and surfactant concentration [30,31,32]. Generally, the tendency to form the simplest spherical micelles is the strongest at the lowest concentrations, above which the self-assembly process starts. For many surfactant solutions and mixed surfactants systems, an increase in concentration causes a change in aggregation numbers and as a result, the micelle phase transition becomes probable. Hence, more scientists interpret the changes in ITC curve as regions of the potential structural rearrangement in micelles [6,33,34,35,36]. Karumbamkandathil and co-workers reported that the inflection points on the calorimetric curves for aqueous solutions of benzethonium chlorides were due to micelle shape transformation [37]. The main purpose of this work is to examine whether the calorimetric titration curves “encode” the structural transitions in aqueous solutions of cationic gemini surfactants, with the use of complementary experimental methods: Small-Angle Neutron Scattering (SANS), Small-Angle X-ray Scattering (SAXS), Scanning Transmission Electron Microscopy (STEM), as well as Monte Carlo simulation. The reason for the choice of alkanediyl-α,ω-bis(dimethylalkylammonium) bromides is motivated by an unusual bell-shaped ITC curve of their aqueous solutions which appears at 308.15–318.15 K after long calorimetric experiments of 4–5 days. Experimental work (ITC experiment with STEM, SANS and SAXS used within a wide range of temperature and concentration) in conjunction with computer simulation allowed us to analyze the process of micellisation and potential shape transitions.

## 2. Results and Discussion

### 2.1. Calorimetric Data

**Figure 1:**
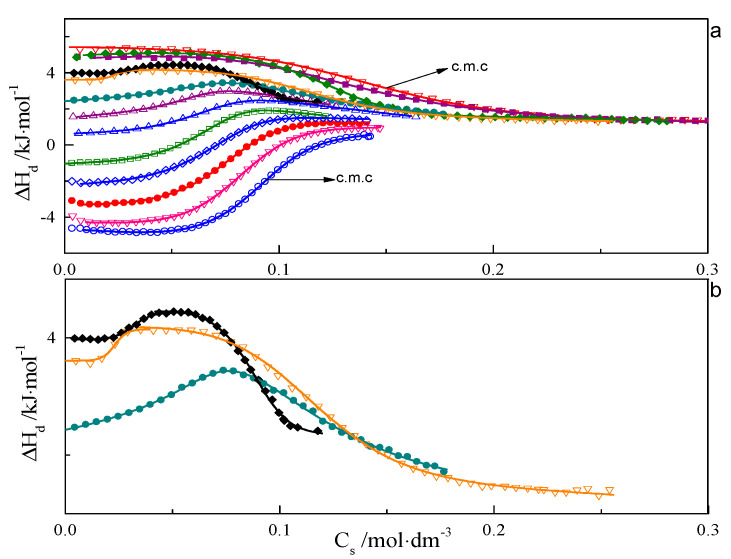
(**a**) Calorimetric titration curve from additions 8-8-8 surfactant to water for temperatures: ○—283.15 K, ∇—288.15 K, •—293.15 K, ◊—298.15 K, □—303.15 K, ∆—308.15 K, ∆—313.15 K, •—318.15 K, ♦—323.15 K, ∇—328.15 K, ♦—333.15 K, ■—338.15 K, ∇—343.15 K. (**b**) Selected calorimetric titration curves for the transition temperatures: •—318.15 K, ♦—323.15 K, ∇—328.15 K. Due to many experimental points, only every fifth point is presented on the plot.

**Figure 2:**
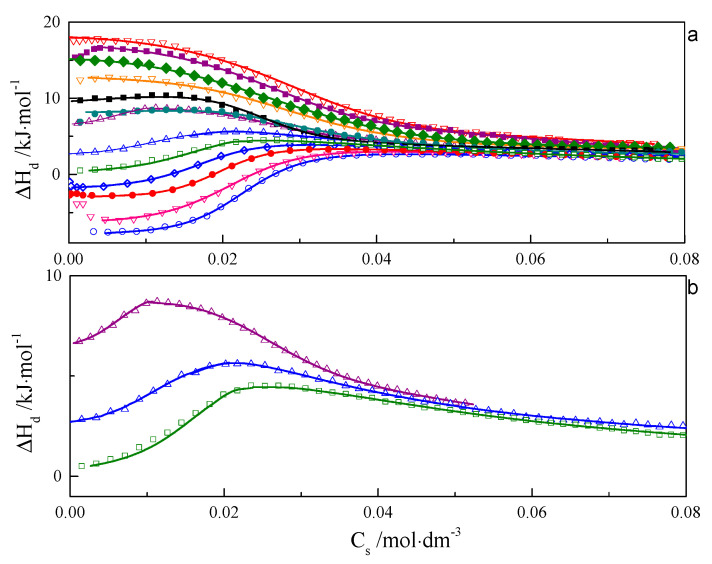
(**a**) Calorimetric titration curve from additions 8-14-8 surfactant to water for temperatures: ○—283.15 K, ∇—288.15 K, •—293.15 K, ◊—298.15 K, □—303.15 K, Δ—308.15 K, Δ—313.15 K, •—318.15 K, ♦—323.15 K, ∇—328.15 K, ♦—333.15 K, ■—338.15 K, ∇—343.15 K. (**b**) Selected calorimetric titration curves for the transition temperatures: □—303.15 K, Δ—308.15 K, Δ—313.15 K. Due to many experimental points, only every fifth point is presented on the plot.

ITC curves for aqueous solutions of surfactants 8-8-8 and 8-14-8 are shown in Figure 1 and Figure 2. All experimental curves for investigated systems wth s = 6, 7, 8, 9 are available in the Supporting Information—Figures S16–S18. For each surfactant system, it was observed that an increase in temperature causes a gradual change in the course of ΔH_d_ = f(C) from endothermic to exothermic. Shapes of the dilution enthalpy curves of surfactant with a spacer with ≤9 carbon atoms for temperature regions: 283.15–303.15 and 328.15–343.15 K are almost sigmoidal with noticeable one inflection point which corresponds to the c.m.c region. For a compound with s = 14, the mentioned temperature regions are shifted and appear at 283.15–298.15 and 318.15–343.15 K. It is worth noting that the micellisation region is less sharp at 343.15 K than at 298.15 K. In the case of the 288.15–303.15 K region, the micellisation occurs at lower concentrations in comparison to that of the 328.15–343.15 K range. The shape of function ΔH_d_ = f(C) for the transition area: 308.15–323.15 K for the investigated surfactant with s ≤ 9 and 303.15–313.15 K in case s = 14 is unusual and resembles two conjoined sigmoidal curves—Figure 1b and Figure 2b.

It is well known that the structures of micelles created at lower concentration often differ in shape and size form with aggregates that appear at higher concentration in 328.15–343.15 K range. For that reason, it is highly probable that ITC curves from the transition area may be interpreted in terms of the first one reflecting the micellisation process and the second one caused by the transformations of aggregates. In order to verify the type of structures created at different concentrations and temperatures, TEM, SANS and SAXS experiments were carried out, as well as computer simulations.

### 2.2. Scanning Transmission Electron Microscopy

**Figure 3:**
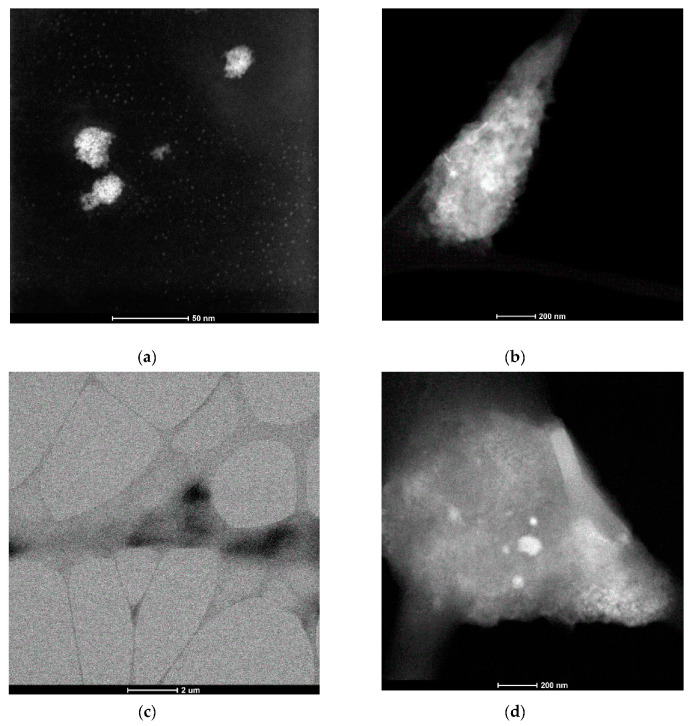
Scanning Transmission Electron Microscopy (STEM) micrographs for aqueous solution of 8-8-8 surfactant (**a**) 0.09 M solution, (**b**) 0.09 M solution after 3 days, (**c**) 0.3 M solution, (**d**) 0.3 M solution—zoom of a region from image (**c**).

The STEM images of the drop-cast solutions of 8-s-8 (s = 6, 7, 8, 9) surfactants were almost the same, and for that reason, we present only the results for the 8-8-8 samples. As is seen in Figure 3a, the two types of structures are visible. At concentration near c.m.c ~ 0.09 M (that corresponds to the inflection point on the ITC curve—Figure 1), the molecules were spontaneously organized into small, approximately spherical shape structures, characterized by diameter in the range 2–3 nm. Bigger agglomerates with diameters of 12–30 nm were observed occasionally. They probably developed during the heating process. Nevertheless, small micelles exhibit a tendency to assemble in groups, which can suggest the mechanism of creating expanded aggregates during heating. It is interesting to find that the morphology of the molecular assemblies in the system changed with time. STEM images for the solution after 3 days exhibit greater structures (10–30 nm)—Figure 3b. For more concentrated solutions, ~0.3 M aggregation into larger structures with an undefined shape dominates—Figure 3c,d. Based on these observations, it is clear that the increasing concentration shifts the equilibrium toward more expanded aggregates for which the shape is difficult to define, but also temperature and time cause the shift into the more complex micelles.

### 2.3. Computer Simulations

**Figure 4:**
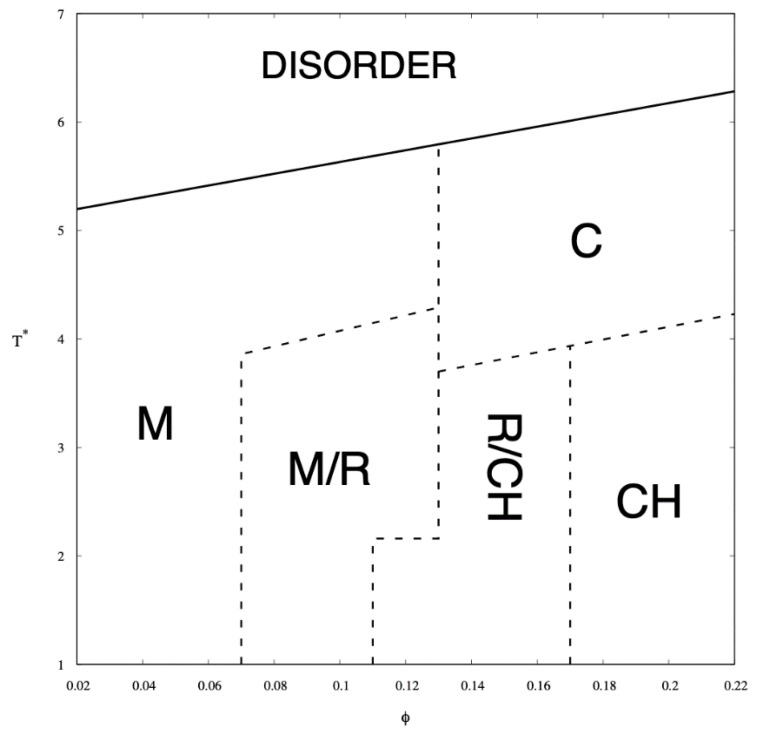
Monte Carlo phase diagram of A8-B2-A6-B2-A8 pentablock colomymer in a selective solvent as a function of the chain volume fraction, φ, and the reduced temperature, T*. Solid line is the approximate critical micelle temperature (CMT)/order–disorder transition (ODT) line while the dashed lines indicate transitions between the following arrangements: micellar (M), rod-like elongated micellar (R), 3-dimensional channels (CH), and cylindrical (C).

**Figure 5:**
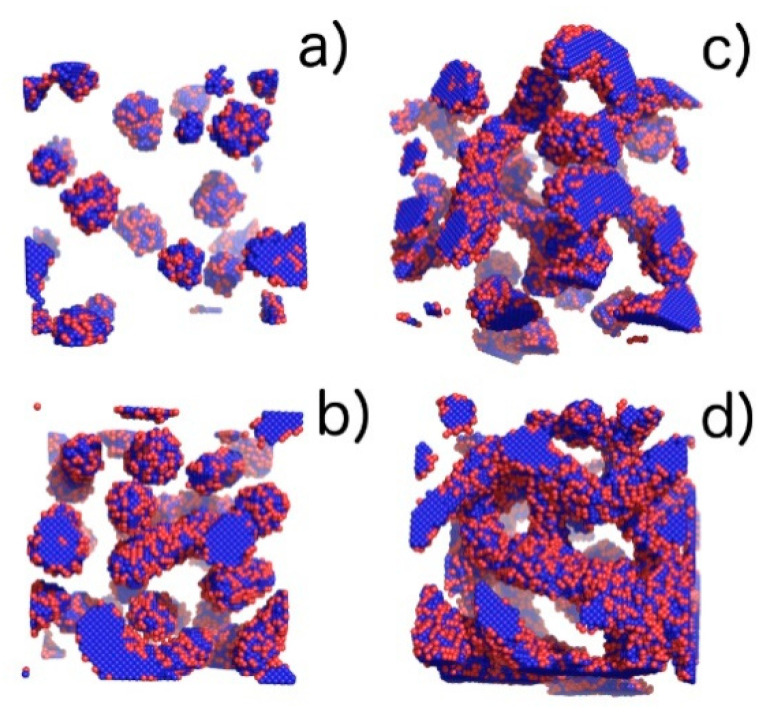
Visualization of phases with the increasing volume fraction (φ) of the copolymer obtained from computer simulations: (**a**) pure micellar phase (M), φ = 0.04, T* = 3.0; (**b**) elongated rod-like micelles (M/R), φ = 0.1, T* = 1.3; (**c**) transition between elongated rod-like micelles and threedimensional Y-channels (R/CH), φ = 0.14, T* = 2.16; and (**d**) pure three-dimensional Y-channels (CH), φ = 0.2, T* = 2.16. Blue colour denotes A-blocks (tails and spacers) while red refer to B-blocks (gemini heads).

Simulations were carried out for ten different copolymer volume fractions, i.e., φ = 0.02, 0.04, 0.06, 0.08, 0.1, 0.12, 0.14, 0.16, 0.18, and 0.2. These concentrations contain the following numbers of chains of length N = 26, 182, 365, 548, 730, 913, 1095, 1278, 1460, 1643, and 1825 respectively. The phases identified by examining the Monte Carlo configurations at all temperatures and copolymer volume fractions are presented in the phase diagram in Figure 4 and illustrated by snapshots shown in Figure 5. The phases identified by examining the Monte Carlo configurations at all temperatures and copolymer volume fractions are presented in the phase diagram in Figure 4 and illustrated by snapshots shown in Figure 5.

**Figure 6:**
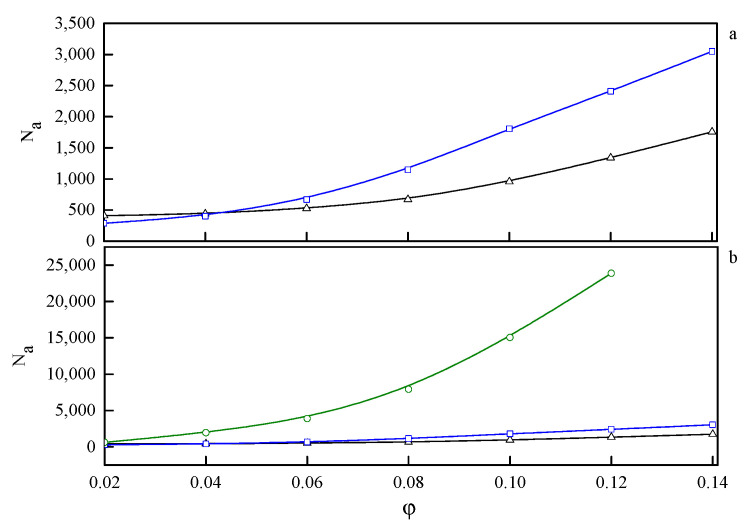
The dependence of the aggregation number, N_a_, on the volume fraction, φ, for three selected temperatures: T* = 1.3 (Δ—triangle), T* = 3.0 (□—squares), and T* = 4.7 (○—circles). On (**a**) the highest temperature is omitted to emphasize the behaviour at lower temperatures. On (**b**) on the other hand N_a_ is on a logarithmic scale for clarity purposes.

In the phase diagram shown in Figure 4, the solid line represents the approximate critical micelle temperature (CMT) and the order–disorder transition (ODT) line. For very low densities, we can talk about CMT, while at higher densities we deal with ODT. Below the CMT/ODT line, a series of phases separated by dashed lines is observed. These lines are approximate due to non-sharp transitions between the individual arrangements. This is the result of discrete values of volume fractions, φ, and temperatures, T*, we simulated, and the narrow φ-range areas where the coexistence of two neighboring phases is observed. In the lowest volume fractions spatially disordered micelles (M) are observed. In a weak segregation limit (WSL), that is, at the temperatures close to the ODT, the micelles shape becomes cylinidrical (C) as the volume fraction exceeds 0.12. At lower temperatures, on the other hand, an increase in volume fraction of the copolymer leads to the micellar phase becoming enriched with elongated rod-like micelles (M/R). As the system reaches φ ≈ 0.12, the ever-increasing proportion of the R phase causes the rodlike micelles to begin to combine and form three-dimensional Y-channels (R/CH). Finally, when the copolymer volume fraction exceeds φ ≈ 0.16, the system turns into the pure three-dimensional Y-channels phase (CH). In Figure 5, we present a selection of representative configuration snapshots showing the above evolution of phases, along with the increasing volume fraction of the copolymer. The blue colour denotes A-blocks (tails and spacers) while red refers to Bs (gemini heads). Towards the increasing φ we observe pure micellar phase (Figure 5a), elongated rod-like micelles (Figure 5b), mix of rod-like micelles and Y-channels (Figure 5c), and pure three-dimensional Y-channels (Figure 5d) respectively. We also report an aggregation number, *N*_a_, specific heat, *C_V_*, mean-squared radius of gyration, $R_{g}^{2}$ and the reduced energy per chain, $\frac{E^{*}}{n_{c}}$, where $n_{c}$ is the number of chains in a given volume fraction. The quantitative dependence of the aggregation number *N_a_* on the volume fraction, φ, and the reduced temperature, T* is illustrated in Figure 6. In Figure 6a, we show only two datasets for the temperatures T* = 1.3 and 3.0 due to emphasize the function course. In Figure 6b, we show in turn *N_a_* on a logarithmic scale for clarity purposes. We can observe that in a weak segregation limit (circles) the micelles are larger than in a low-temperature strong-segregation limit (triangle).

**Figure 7:**
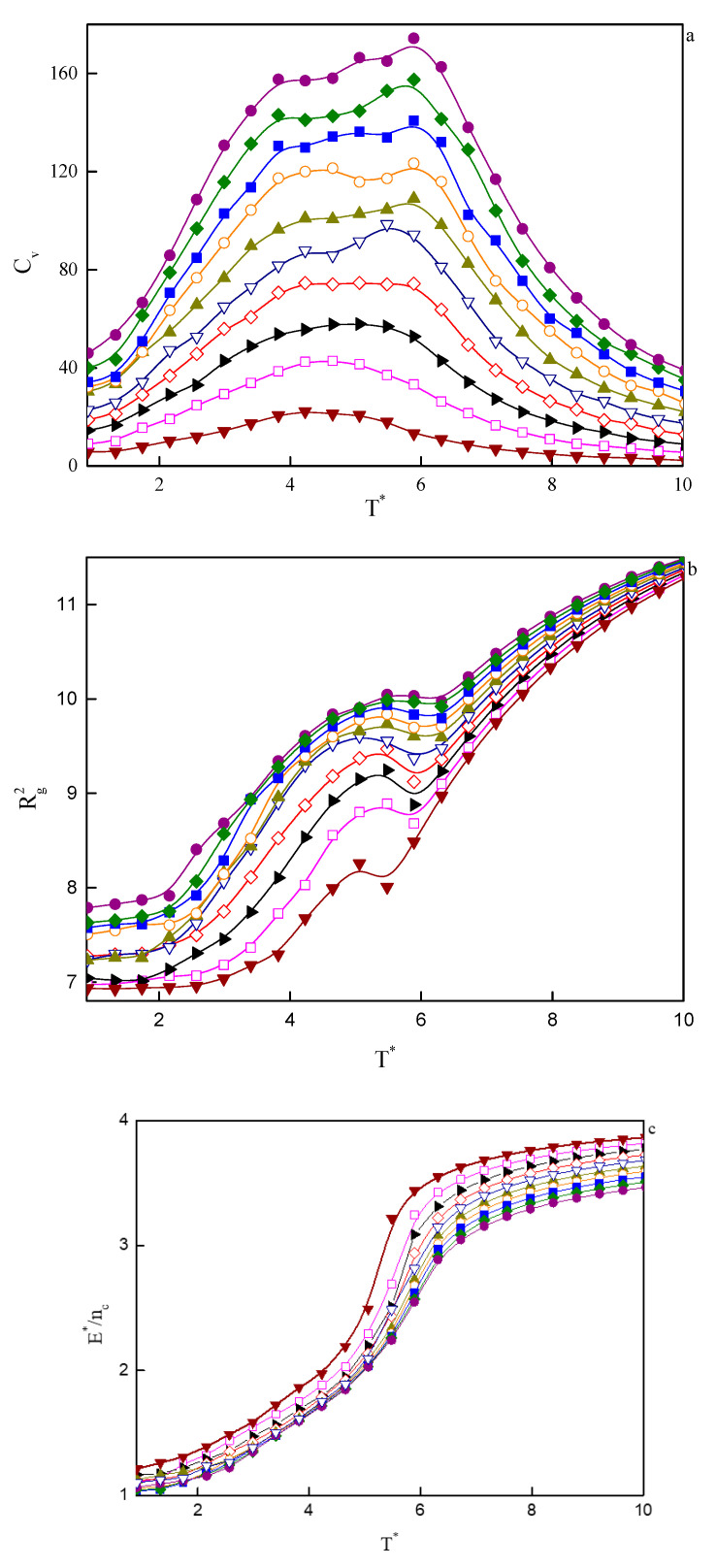
Temperature dependencies of selected properties for a series of copolymer volume fractions, φ, from computer simulations: (**a**) specific heat, *C_V_*; (**b**) mean-squared radius of gyration, $R_{g}^{2}$; (**c**) reduced energy per chain,$\;\frac{E^{*}}{n_{c}}$.

In Figure 7, the temperature dependence of the specific heat (a), the mean-squared radius of gyration (b), and the reduced energy per chain (c), for a series of considered copolymer volume fractions are plotted. At the lowest volume fractions, that is, 0.02 < φ < 0.04, we observe a single maximum in specific heat at the temperature, which we identify as the critical micelle temperature, T*_CMT_. We are talking here about CMT rather than ODT because micelles in such small copolymer volume fractions are disordered (Figure 5a). When we turn our attention to Figure 7c, we see that the aggregation process is accompanied by a significant drop in energy, as well as a decrease in the mean-squared radius of gyration (Figure 7b). At higher φ’s, the maximum broadens and, starting from φ ≈ 0.1, can be considered as two broad overlapping maxima. The ones on the high-temperature side denote ODT, while the maxima at lower temperatures are associated with the transition from one type of order to another one (for example, three-dimensional Y-channels turn into cylinders). The process of changing the type of order is not sharp and runs continuously, so the maxima in the specific heat are also very wide. It is notable that the energy per chain in lower copolymer volume fractions is higher than the energy of systems with higher φ’s (Figure 7c). This becomes clear when we realize that energy is related to the interface between hydrophobic tails/spacers and the solvent. At low φ’s, due to the small size of aggregates, the number of monomers per chain that will have contact with solvent molecules will be higher than in the larger concentrations. In higher concentrations, on the other hand, aggregates are larger and statistically most monomers of tails and spacers will be located inside the aggregates without direct contact with the solvent.

### 2.4. Small-Angle Neutron Scattering and Small-Angle X-ray Scattering

**Figure 8:**
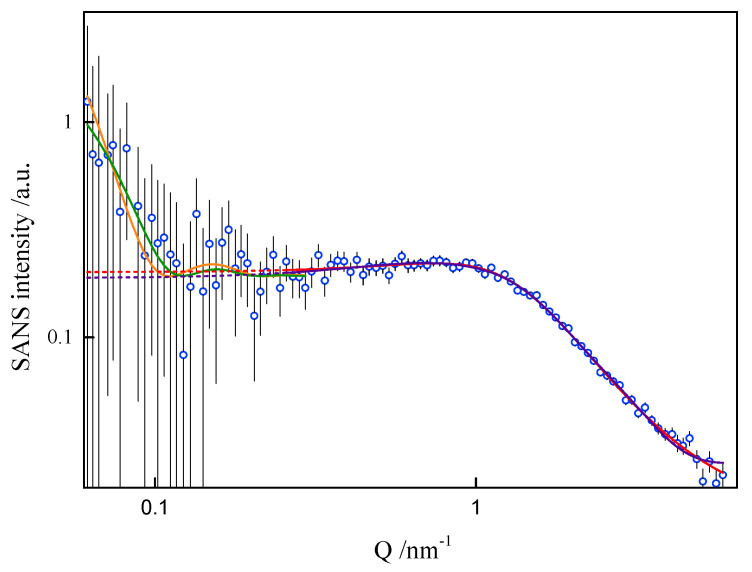
Small-angle neutron scattering from the 8-6-8 aqueous solution at 25 °C. Fit to data of four models, two models in low-Q and two in high-Q ranges, performed in low-Q and high-Q ranges separately: —lamellae, —polyelectrolyte, —charged ellipsoid, —fuzzy sphere. Dashed lines are extrapolations of mid- and high-Q models towards the low-Q end (not fitted in this region).

In order to assess the shape and size of gemini surfactant particles in an aqueous solution, the small-angle scattering (SANS) measurements were performed for the group of 8-s-8 (s = 6, 7, 8, 9, 14) surfactants. A typical SANS signal is shown in Figure 8. It corresponds to the D_2_O solution of the 8-6-8 micelles at 298.15 K.

**Figure 9:**
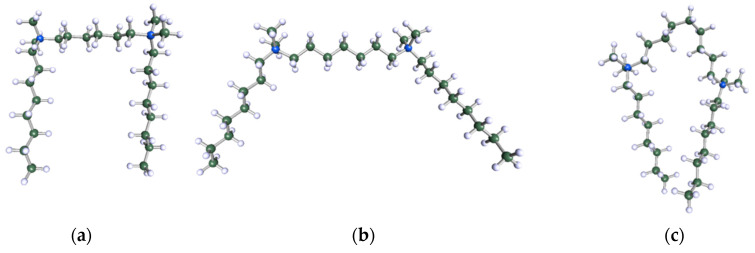
DFT-optimised geometries of the molecules: (**a**) 8-6-8, (**b**) 8-7-8 and (**c**) 8-9-8.

**Figure 10:**
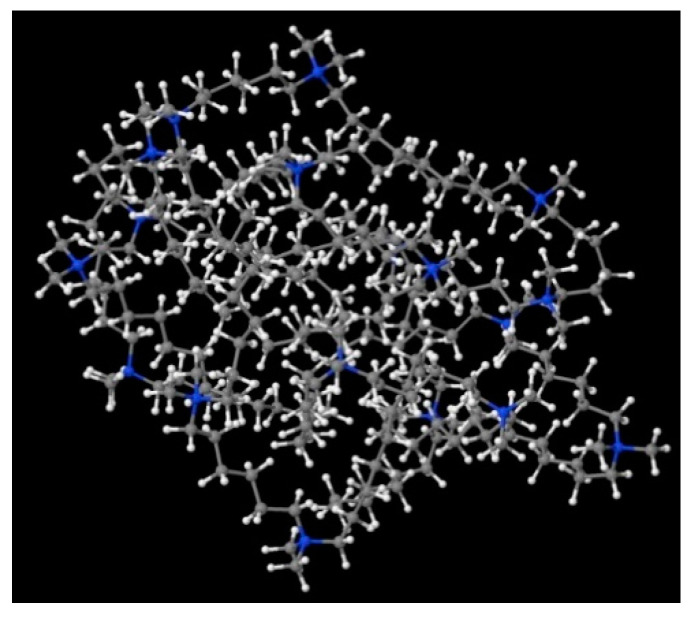
A possible micelle of nine 8-6-8 molecules as optimised by means of semiempirical PM7 approach, as implemented in Mopac.

None of the well-known standard model shapes can satisfactorily reproduce this sort of SANS signal over the whole range of momentum transfer, see Figure 8. This is not a surprise. Very often, SANS signals are treated piece-wise by different models, as different features of real scattering particles show up in different sub-ranges of momentum transfer attainable in the experiment. It is just a manifestation of the complexity of real-life scattering objects as compared to idealized theoretical models. The micelles under the present study are composed of odd-shaped (“H”-shaped) charged molecules, which cannot be packed in a simple “hydrophobic-in, hydrophilic-out” manner, as “well-behaved” rod-like amphiphiles can. The micelles of such surfactants are sometimes considered core–shell objects, with the shell composed of hydrophilic heads and the core made of hydrophobic tails, effectively squeezing the solvent out of their volume. The micelles of gemini surfactants are, on the other hand, highly non-uniform, with possible irregular voids in their internal structure and extremely jagged exterior (see Figure 9 and Figure 10). In general, although they appear to be globular, nearly spherical, lacking measurable core–shell characteristics, they are intractable in terms of standard model form factors.

In order to understand the possible structure of a gemini surfactant micelle, simulations were performed using a semi-empirical PM7 approach as implemented in the Mopac program [38]. Input micelles were composed of molecules, whose geometries had been previously optimised by the Density Functional (DFT) method (triple-zeta basis def2-TZVPPD, B3LYP hybrid functional with Caldeweyer–Grimmie D4 dispersion corrections), taking into account the cationic character of the surfactant “heads” [39]. Such geometries are usually non-unique, as, e.g., various conformations are possible within eight-carbon terminal chains, and this was taken into account in the simulations. Gemini surfactant molecules also exhibit a clear odd–even effect with respect to the length of the spacer chain, as shown in Figure 9. Moreover, a longer spacer facilitates other than “all-trans” conformations thereof, enabling hydrophobic attraction of terminal chains. It is now clear that whatever aggregates are formed in the solution, it is not possible that all tails point inwards, with “heads” organized in a kind of shell. A sample micelle composed of nine 8-6-8 molecules is shown in Figure 10.

Optimisation was performed in a simulated aqueous environment, taking into account the solvation effect within the “Conductor-like Screening Model”—COSMO [40]. The results of such a quantum chemical COSMO calculation are summarized in Table 1.

**Figure 11:**
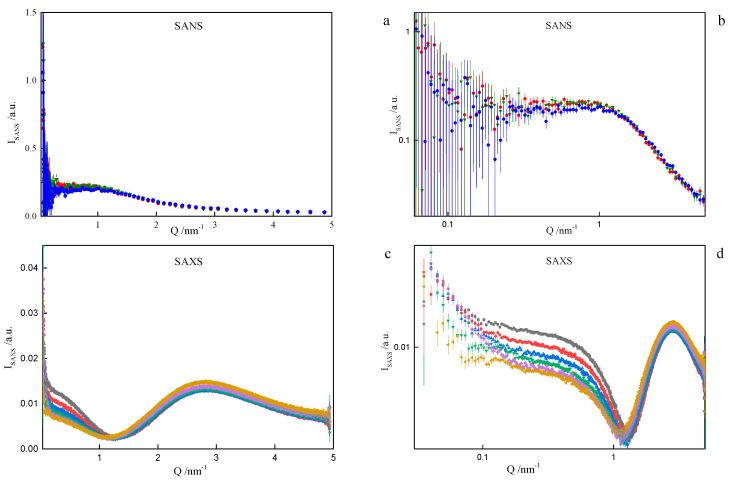
Observed Small-Angle Neutron Scattering (SANS) (**a**) and (**b**) and X-ray Scattering (SAXS) (**c**) and (**d**) spectra for the cationic dimeric 8-6-8 surfactant system for C_s_ = 0.22 mol dm^−3^ at: ■—293.15 K, ●—298.15 K, ▲—303.15 K, ▼—308.15 K, ♦—313.15 K, ◄—318.15 K.

On the basis of the results listed in Table 1, it can be concluded that surfactants with longer spacer exhibit the tendency to create larger-in-volume and less cohesive aggregates. This arises from the spacer rigidity and electrostatic interactions, as well as due to volume limits of the micelle core. Both the formation and behaviour of gemini surfactant micelles in solution are strongly affected by the ionic character of the species and, consequently, by the presence of counterions in the ambient environment. Not only was this was found during numerical simulations, but also while attempting to model-fit the SANS data. The software used for that purpose was SasView [39]. The best description of the SANS data in the middle- and high-Q region was obtained by assuming a model relevant to a polyelectrolyte solution, as shown in Figure 8, even though the matter under study was by no means a polymer electrolyte. Consequently, the fitted model parameters, although meaningful, had no direct correspondence to the gemini surfactant micelles. An almost equally good fit to data could be provided by a charged ellipsoid model of Hayter and Penfold as implemented in [41] and referenced in its documentation. Both models assume that an important role is played by electrostatic interactions in the system, which is the case with charged gemini surfactant micelles suspended in water in presence of counterions. The low-Q region, however, exhibits an up-turn of SANS intensity, whose adequate description would require the turn to much lower momentum transfers, possibly to the USANS domain. On the SANS instrument used in the present work, the low-Q region is affected by large experimental uncertainties and it can only be treated in a qualitative manner. At this point, however, we can safely assume that the low-Q up-turn is caused by aggregation of the micelles. Such an aggregation might possibly be mediated by the presence of counterions in the solvent. Two ways that micelle aggregates were considered were globular, leading to a fuzzy sphere, and lamellar. Both approaches could satisfactorily reproduce experimental data in the low-Q region, as shown in Figure 8. The resultant characteristics of such superstructures were meaningful (e.g., the diameter of a fuzzy sphere of 72 nm, or thickness of the lamellae about 57 nm). Some more insight into the morphology of the micelles can be gained from the SAXS experiment. Small-Angle X-ray Scattering (SAXS) is a technique that measures fluctuations of average electronic density. For comparison, small-angle neutron scattering (SANS) responds to fluctuations of neutron scattering length density. Both are, therefore, contrast-based methods. If the scatterers (scattering particles) are monodisperse and homogenous, SAXS can deliver reliable information on basic scatterer characteristics such as shape, radius of gyration, maximum dimension or pair distribution function. Strictly speaking, the above conditions are nearly never fully satisfied. SAXS provides measures to determine (to some extent) to what degree the particles and “non-ideal”, e.g., flexible or polydisperse. Under some circumstances (not necessarily at high concentrations) the particles can aggregate to form pairs or greater superstructures. From small-angle scattering, we can infer whether this is the case. Both SANS and SAXS intensities exhibit a significant up-turn at the lowest Q accessible to the instruments used, see Figure 11.

This most likely indicates that the micelles aggregate into larger superstructures, but still remain distinguishable particles. As the temperature rises, the micelle aggregates either decompose or swell. Should the latter happen, the upturn would move towards smaller Q, out of our experimental window. In order to study this process, an ultra small-angle experiment (USANS, USAXS) would be needed. Our SAXS data were processed in a batch mode by the ATSAS suite of programs a standard approach used in the study of biological samples [42]. In the Guinier region, the SAXS intensity can be approximated by:

(1) $$I\left(Q\right)=I\left(0\right)exp\left(-\frac{Q^{2}R_{g}^{2}}{3}\right)$$

**Figure 12:**
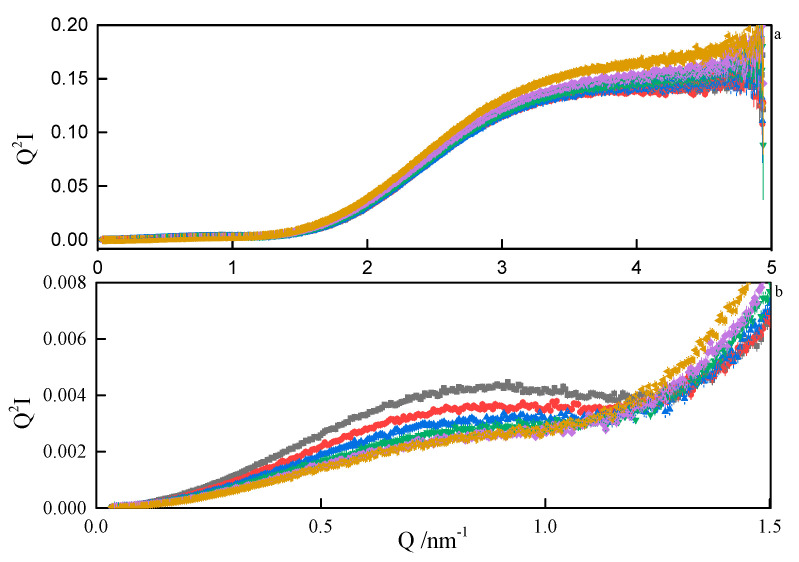
Kratky plots (**a**) and zoom (**b**) from SAXS analyses of 8-6-8 surfactant system at C_s_ = 0.22 mol dm^−3^ at: ■—293.15 K, ●—298.15 K, ▲—303.15 K, ▼—308.15K, ♦—313 K.15, ◄—318.15 K.

Therefore, from the linear parts of the Guinier plots $\left(Q^{2}I\left(Q\right)\;vs.Q\right)$, the radii of gyration of the micelles could be estimated. The values ranged between 1.6 nm and 1.9 nm, depending on the spacer length. Since the micelles are likely to form superstructures by the grouping of individual particles, the estimated *R_g_* should be treated with decreased confidence. The values of the product ${\left(QR_{g}\right)}_{max}$ are convenient indicators of the scatterer particle shape. If $0.9<{\left(QR_{g}\right)}_{max}<1.3$, the particles are globular, if less than 0.8, they are elongated [43]. The values obtained in our work ranged between 1.2 and 1.3, meaning that the micelles are globular. The same conclusion was drawn from the analysis of SANS data, where any attempt to model-fit an ellipsoidal shape ended with the resultant axes being equal. Additionally, the general shape of the SAXS data plot (Figure 11) is commonly attributed to the nearly-spherical particles [44]. If a spherical shape of the micelles is assumed, then the real dimensions of the spheres can be estimated from the theoretical relation $R_{g}^{2}=\frac{3}{5}R^{2}$ [45]. Some more insight into the micelle properties can be obtained from the Kratky plots $\left(Q^{2}I\left(Q\right)\;vs.Q\right)$. A sample Kratky plot is shown in Figure 12. At lower temperatures, a nearly-bell-shaped plot around *Q* = 0.08 nm^–1^ indicates a more compact scattering particle. As the temperature rises, more flexibility within the micelles is seen.

**Figure 13:**
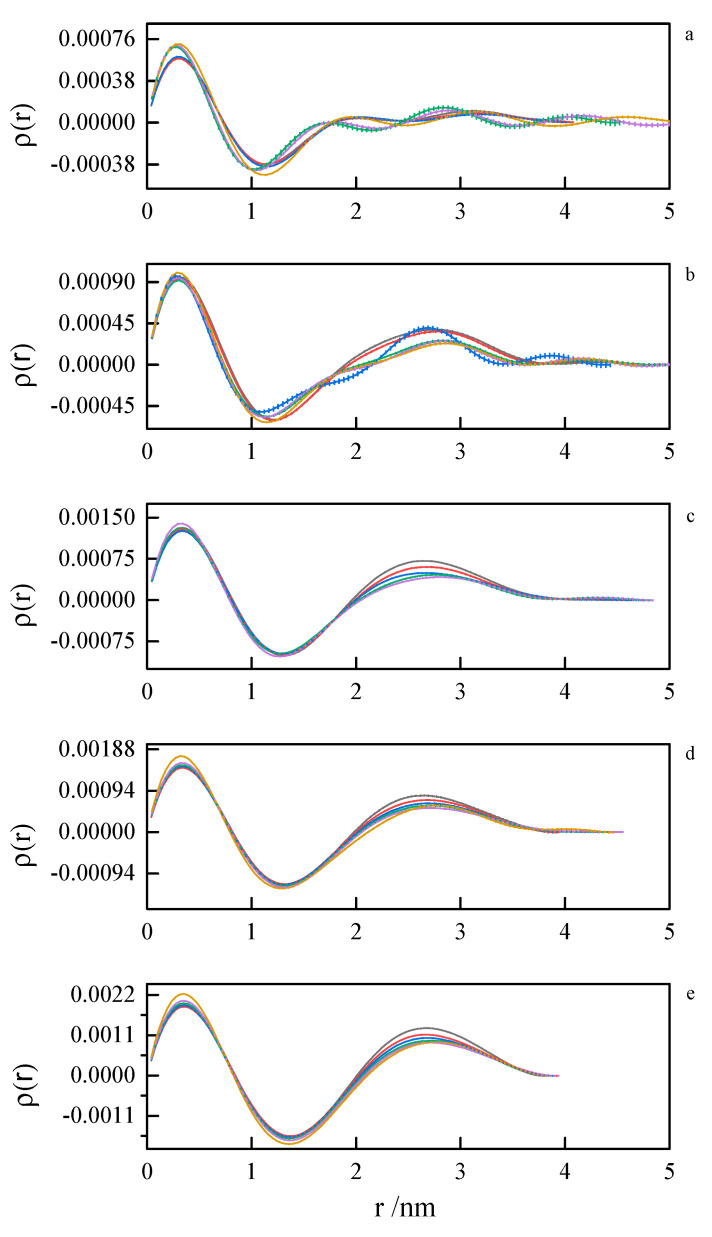
Pair distance distribution functions derived from SAXS data by indirect Fourier transform for 8-6-8 surfactant system at concentrations (**a**) 0.098 mol dm^−3^, (**b**) 0.112 mol dm^−3^, (**c**) 0.149 mol dm^−3^, (**d**) 0.185 mol dm^−3^, (**e**) 0.222 mol dm^−3^ at 293.15 K, 298.15 K, 303.15 K, 308.15 K, 313.15 K, 318.15 K.

**Figure 14:**
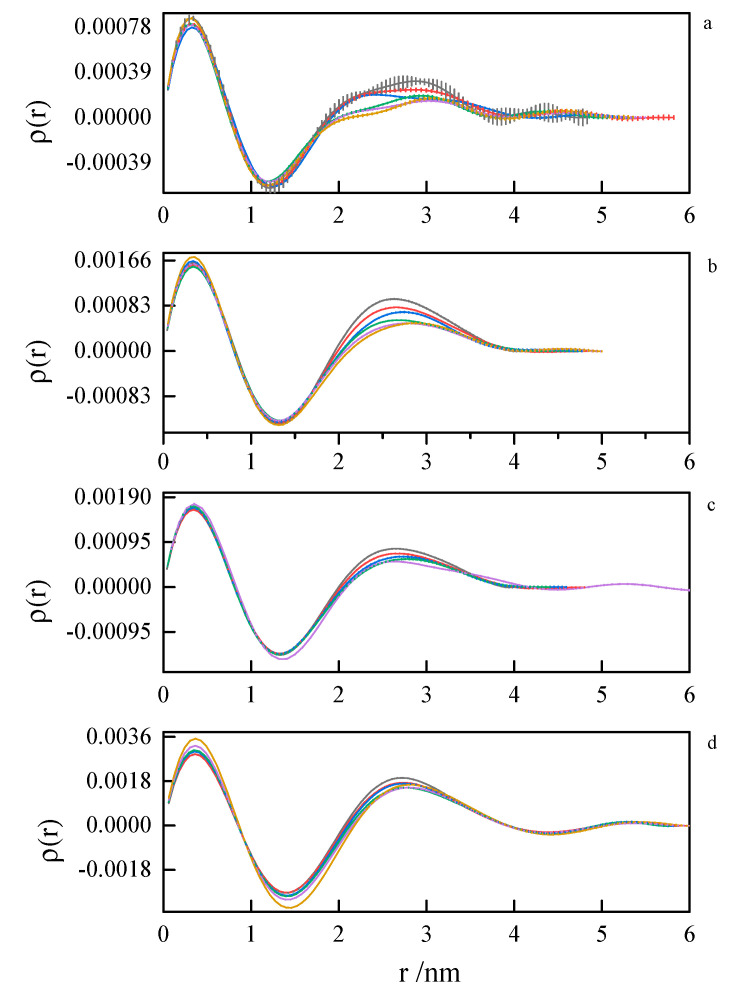
Pair distance distribution functions derived from SAXS data by indirect Fourier transform for 8-8-8 surfactant system at concentrations (**a**) 0.104 mol dm^−3^, (**b**) 0.171 mol dm^−3^, (**c**) 0.197 mol dm^−3^, (**d**) 0.342 mol dm^−3^ at 293.15 K, 298.15 K, 303.15 K, 308.15 K, 313.15 K, 318.15 K.

Pair distance distribution functions (PDDF) were derived from SAXS data by indirect Fourier transform (IFT), as implemented in the GNOM program from the ATSAS suite. Due to a very similar tendency, only the results for 8-6-8 and 8-8-8 systems were presented—Figure 13 and Figure 14. The obtained results for other investigated systems are summarized in Supporting Information Figures S19 and S20.

The first peak in the PDDF curves corresponds to the intramolecular spatial correlations. At small surfactant concentrations (where first inflection on the ITC curve appears), we can see multimodal PDDF curves, clearly corresponding to structures other than globular micelles, most likely building from the aggregating of smaller clusters. Should this be true, we can speculate about the micelle formation process. A transition to regular, unimodal PDDFs between 1.5 and 4.0 nm indicates the completion of the micellization process. Once formed, the micelles swell and become less compact as the temperature rises. This is concluded from the PDDF curves broadening with temperature Figure 13 and Figure 14.

### 2.5. Interpretation of ITC Curves and Thermodynamics of Micellisation

Taking into account the visualization of self-assembly, as well as experimental results (TEM, SANS, SAXS), it was shown that investigated systems exhibit the tendency to create quite small structures as a dominative form at low concentration. An advanced morphology of aggregates appears in a more concentrated solution. The less sharp inflection on the curves form ranges 328.15–343.15 K and 318.15–343.15 K for s = 14, representing the region where a more expanded structure dominates. This can be explained by the meaningful thermal movements of molecules which lead to the partial breakdown of the three-dimensional structure of water around nonpolar parts of amphiphiles, and consequently, hydrophobic interactions become weaker and hinder the self-assembly [46]. Hence, creating simple aggregates at lower concentrations for the temperatures 328.15–343.15 K is unfavorable. It follows from our ITC data from the obtained SANS and SAXS results and simulations that, in the range of higher concentrations and at higher temperatures, the aggregates undergo structural changes affecting their shapes. The corresponding ITC curves can therefore be interpreted in terms of two conjoined sigmoidal curves, the first one reflecting the micellisation process and the second one caused by the transformations of aggregates. Hence, both processes would then be characterized by a non-zero enthalpy and the inflection points corresponding to the c.m.c value and concentration of aggregate transition (we name C_trans_) respectively. The determined values of critical micelle concentration and micelle transition concentration for each of the examined systems within a wide temperature range are summarized in Table 2 and Supplementary Materials Tables S1–S4. The thermodynamic quantities (collected in Table 2 and Supplementary Materials Tables S1–S4) show that the process of creating aggregates, as well as the shape transition process, is spontaneous (ΔG < 0) and entropy-driven in all temperature ranges.

It is typical for the system investigated that a temperature increase causes the value of micelle formation and enthalpy of micelle transition to decrease. The negative value of enthalpy confirms that the contribution of London dispersion interactions are crucial and these attractive forces facilitate the micelle transition and micelle formation, which is especially visible at a higher temperature. The gain of the exothermic value of $\Delta H_{m.trans}$ suggests a tendency to reduce the number of nonpolar parts exposed to the solvent and in consequence to create more complex structures. It was observed that the presence, as well as the position of the second inflection, is correlated with the spacer chain length. Generally, for longer spacer chains, the bell-shaped curves appeared at lower temperatures and similarly, as with the c.m.c region, the second inflection emerged at a lower concentration in comparison with surfactants with shorter spacers. The effect of the structure of the surfactant molecule—in particular, spacer chain length—on the aggregation properties is also reflected in the thermodynamic potentials. More negative values of: $\Delta G_{m}$*,* $\Delta G_{m.trans}$*,* $\Delta H_{m.trans}$*,* and $\Delta H_{m}$ at higher temperature ranges for molecules with s ≥ 9 indicate a stronger tendency for aggregation as well as a trend of micelle growth. These observations indicate that the aggregation abilities are determined by interaction among the molecules, conformational entropy of the spacer and geometrical effects of lengthening spacer or an odd or even number of carbon atoms in the spacer [47,48,49]. The calculated energies for conformations of single molecules with spacer s = 6, 7, 8, 9 in aqueous solution were presented in Table 3.

It is notable that spacer elongation causes an energetic gain equal to 0.17 × 10^−15^ J per CH_2_ unit in the spacer chain. The lack of energy differences for gauche and trans conformations suggests their coexistence in diluted solutions (dispersed state). Therefore, a change of monomer conformation must occur at higher concentrations and affects the aggregation process [47]. Furthermore, the odd number of carbon atoms in the spacer probably influences the possibility of the conformational change and hinders the aggregation and transition processes for the same reasons. For molecules with longer and more flexible spacers, the chain usually adopts folded or looped configurations due to the contact with a polar solvent. Nonetheless, during the aggregation and transformation in the micellar solution, the hydrophobic spacer incorporates into the micelle core more efficiently which in turn reduces contact with water and supports the aggregation process. This is well visible, especially in the case of a surfactant with s = 14 for which the micellisation and transformation parameters are the most favourable.

## 3. Experimental Section

### 3.1. Materials

Hexanediyl-α,ω-bis(dimethyloctylammonium) bromide 8-6-8, heptanediyl-α,ω-bis(dimethyloctylammonium) bromide 8-7-8, nonanediyl-α,ω-bis(dimethyloctylammonium) bromide 8-9-8, dodecylediyl-α,ω-bis(dimethyloctylammonium) bromide 8-14-8 were synthesized according to the procedure described in the literature [50]. The obtained surfactants were recrystallized five times to get the purity ≥98% and then dried under vacuum for a minimum of 72 h at 70 °C. Details are presented in the Supplementary Materials Figures S1–S10. The amount of water was controlled by the Karl Fischer method. Due to the hygroscopicity of surfactants, each solution was prepared in a vacuum dry box. Aqueous solutions of surfactants investigated for ITC and X-ray scattering measurement were prepared with Milli-Q water (MERCK), and were kept away from the light. In the case of SANS measurement, D_2_O (99.994 atom% D, Sigma Aldrich, Poland) was used instead, in order to enhance contrast.

### 3.2. Methods

#### 3.2.1. Calorimetric Measurements

The Microcal VP ITC calorimeter was used during the experiments. The concentrated aqueous solution of surfactant was injected from a 240 µL syringe in appropriate aliquots at an interval of 20 min into the measuring cell (1.4 mL) filled with Milli-Q water. The solution was then stirred at a constant speed of 307 rpm. The measurements were performed at: 283.15 K, 288.15 K, 293.15 K, 298.15 K, 303.15 K, 308.15 K, 313.15 K, 318.15 K, 323.15 K, 328.15 K, 333.15 K, 338.15 K, 343.15 K. The time for one experiment was 4–5 days. The obtained enthalpograms were described by Modified Boltzman Equation [28,51].

(2) $$\Delta H_{d}=\Delta H_{d\left(f\right)}{\left[1+\left(\frac{\Delta H_{d\left(i\right)}-\Delta H_{d\left(f\right)}}{\Delta H_{d\left(f\right)}}\right)\left(1+\frac{\exp(C_{s}-C_{c.m.c}}{\Delta C_{s}}\right)\right]}^{-n}$$

where:$\;C_{s}$ is the surfactant concentration in the system, $\Delta C_{s}$ is the constant interval of surfactant concentration, indices i and f describe the stages: initial and final respectively, $C_{c.m.c}$ and n are fitted parameters.

The symbols: $\Delta H_{d\left(f\right)}$ and $\Delta H_{d\left(i\right)}$ represent functions of pre- and post-micellar regions of titration curve:

(3) $$\Delta H_{d\left(i\right)}=f\left(C_{s}\right)=H_{ia}+H_{ib}C_{s}$$

(4) $$\Delta H_{d\left(f\right)}=f\left(C_{s}\right)=H_{fa}+H_{fb}C_{s}$$

where: $H_{ia}$, $H_{ib}$, $H_{fa}$, $H_{fb}$ are fitting parameters.

The enthalpy of micellisation was obtained from Equation (5):

(5) $$\Delta H_{mic}=\Delta H_{d\left(f\right)}\left(C_{c.m.c}\right)-\Delta H_{d\left(i\right)}(C_{c.m.c})$$

The critical micelle concentration value was obtained as a zero of the second derivative of enthalpy as a function of concentration:

(6) $$\frac{\partial^{2}\Delta H_{d}}{\partial C_{s}}\left(C_{c.m.c}\right)=0$$

Other thermodynamical functions of micellisation were calculated according to the model for ionic surfactants taking into account the degree of counterion binding β estimated from the conductivity data [52,53]:

(7) $$\Delta G_{mic}=\left(0.5+\beta \right)RT\;ln\left(X_{c.m.c}\right)$$

and by using the Gibbs Helmholtz’s relation:

(8) $$\Delta S_{mic}=\frac{\Delta H_{mic}-\Delta G_{mic}}{T}$$

#### 3.2.2. Conductometric Studies

The conductivities measurements for surfactants were carried out with an automatic bridge for conductivity measurement type 6440 B from Wayne Kerr. The operating frequency of the bridge was 1 kHz. Temperature dependence of the degree of counterion binding β allowed to calculate the $\Delta G_{mic}$ for ionic surfactants [52]. Obtained results of β parameters were collected in the Supplementary Materials Figures S11–S15.

#### 3.2.3. Small-Angle Neutron Scattering Measurements

The SANS experiments were carried out by means of the small-angle time-of-flight diffractometer YuMO at the Frank Laboratory of Neutron Physics (FLNP) of the Joint Institute for Nuclear Research (JINR) in Dubna, Russia [54,55]. The instrument can accept incident neutrons of 0.5 < λ < 8 Å. With its system of two PSD detectors located at 4.5 and 13 m from the sample position, it covers the Q range of 0.0063 Å^−1^ to 0.58 Å^−1^. Each dataset was collected for up to two hours of measurement time to achieve sufficient statistics. The surfactant solutions with concentrations 1.1 × c.m.c-5 × c.m.c (determined by means ITC at 288.15 K) were prepared in heavy water (Sigma Aldrich 99.994 atom% D). The prepared samples were placed into 1 mm thick quartz cuvettes. Background scattering was subtracted by comparison with a corresponding pure 100% D_2_O sample. The SANS measurements were performed within the temperature range of 283.15–333.15 K with the accuracy of at least ±0.1 K in standard thermobox, controlled by the “Lauda” thermostat [56]. The raw data were converted into absolute intensities after standard corrections for background and transmissions using SAS program [57].

#### 3.2.4. SAXS Measurements

SAXS measurements were conducted at Moscow Institute of Physics and Technology, Dolgoprudny, Russia on a Rigaku SAXS instrument with a pinhole camera attached to a rotating Cu anode X-ray high-flux beam generator (MicroMax 007-HF) which operated at 40 kV and 30 mA (1200 W) [58]. A multiwire gas-filled area detector Rigaku ASM DTR Triton 200 (diameter 200 mm, pixel size 200 μm) was placed at a distance of 203 cm from the sample. The entire optical path of the X-ray beam was kept in vacuum during data collection. The detector covered Q-range of 0.005–0.5 Å^−1^ (*Q* = 4*π sinθ*/*λ*, where λ is the wavelength and 2θ is the scattering angle). The beam size was 400 μm and the exposure time ranged from 1800 s to 2500 s. The samples were placed in a temperature-controlled holder and SAXS data were collected at: 293.15 K, 298.15 K, 303.15 K, 308.15 K, 313.15 K, 318.15 K, 323.15 K. The samples were incubated for 30 min before collecting data at each temperature in the heating direction.

#### 3.2.5. Scanning Transmission Electron Microscopy

STEM images were performed by a Phillips CM 30 (source LaB6). Aqueous solutions of 8-6-8 and 8-7-8 surfactants differ with concentration (C = 0.09 mol dm^−3^ and C = 0.295 mol dm^−3^) were prepared three days before investigations. The prepared solution was drop casted onto a TEM Cu grid coated with a carbon foil, and subsequently heated up to 323.15 K and dried. The results of self-assemblies in solution were observed in the scanning transmission electron microscopy (STEM) mode using the high angular annular dark field (HAADF) detector on a FEI Tecnai Osiris operating at 200 kV with a resolution of 0.15 nm in STEM. The FEG instrument was equipped with a unique in-column EDX system allowing fast acquisition of EDX maps of the sample.

#### 3.2.6. Computer Simulations

The Monte Carlo simulations were performed using simple motion algorithm (SMA). This model is based on the Face Centered Cubic (FCC) lattice with coordination number z = 12 and bond length equal to a √ 2, where a is the lattice constant. Since in real systems monomers vibrate around their equilibrium positions, statistically the length of a bond can be considered constant, and in the SMA model bonds were not allowed to be stretched or broken. Standard periodic boundary conditions were also imposed. A single gemini molecule was modelled as A8-B2-A6-B2-A8 pentablock copolymer, where A blocks denote hydrocarbon segments while B blocks refer to the gemini heads. Subscript denotes the number of monomers in a given block. The coarse-graining procedure was chosen so that a single A-monomer refers to a single CH_2_ group, while two B-monomers replace a single head of the gemini molecule. The size of the simulation box, chosen to completely accommodate three stretched chains, was 78 × 78 × 78, and the lattice was completely filled with chain segments (A or B) and solvent (S). In order to model tails and spacers as solvophobic and heads as solvophilic, the interaction energy between species i and j (i, j = A, B, or S) was taken as ε_ij_ with ε_AA_ = ε_BB_ = ε_SS_ = ε_BS_ = 0 and ε_AB_ = ε_AS_ = ε = χkT/(z − 2), where χ is the Flory parameter, k is the Boltzmann constant, T denotes absolute temperature, and z(=12) is the lattice coordination number. Having ε as an energy unit we can define the reduced energy and the reduced temperature as:

(9) $$E^{*}=\frac{E}{\varepsilon }$$

(10) $$T^{*}=\frac{kT}{\varepsilon }$$

In the Monte Carlo approach, the standard Metropolis algorithm generates a random set of points in configuration space which resemble the distribution of such points in thermal equilibrium. This method works well at high temperatures, while it traps the system at local free energy minima at low temperatures. To alleviate the problem, we used the parallel tempering (PT) protocol [59,60], in which parallel simulation of many replicas in the relevant temperature range was performed so that the energy barriers between local free energy minima could be effectively overcome. We simulated in a set of 72 temperatures and started each simulation by equilibrating the system in the athermal limit for which ε/(kT) = 0. When the chains adopted statistical conformations and random orientations the system was considered to have reached thermal equilibrium. The athermal systems were equilibrated for 3 × 10^6^ Monte Carlo steps (MCS) and a single MCS is defined as a statistical attempt to move all the chain segments in the system. Athermally equilibrated configurations were then thermally equilibrated with PT protocol for 3 × 10^6^ MCS, followed by 2 × 10^6^ MCS for collecting the data. In the data collection stage, the PT protocol was turned off so that the neighbouring replicas did not exchange between themselves. The simple motion algorithm, in contrast to the cooperative motion algorithm (CMA) [60,61,62,63] which is extremely effective for dense melted polymers, is very efficient in highly diluted polymer solutions.

## 4. Conclusions

There is no doubt that ITC is an indispensable method that can be used to describe the aggregation process of a surfactant system from the thermodynamic point of view. However, the less obvious ITC curve shapes, along with their unexpected changes and behaviour, should provoke scientists into the in-depth analysis of the nature of aggregation supported by structural techniques or theoretical calculations. In this study, we conclusively proved that the shape of ITC curves reflects the structural reorganisation of the micelle shape that we confirmed by using transmission electron microscopy structural techniques (SANS and SAXS) as well as by theoretical calculation. On the basis of the results obtained, we can conclude that in a specified temperature range, the cationic gemini surfactant undergoes structural changes which affect their shapes. The temperature range of the micelle transformation strictly depends on the surfactant spacer chain length. Surfactants with longer spacers exhibit structural transition at lower temperatures and as a result, the tendency to create more expanded micelles is stronger. This can be explained by the increase in the overall hydrophilicity of the molecule and incorporation of the spacer the micelle core. Furthermore, aggregates formed at higher temperatures appear at very high concentration, and for that reason, their shape is branched, less compact and difficult to categorize. It was found that the morphology of the molecular assemblies in the system changed with time as confirmed by the ITC curves and STEM images.

## Figures and Tables

**Table 1:** Micelle parameters obtained from simulations based on Conductor-like Screening Model (COSMO).

	8-6-8		8-7-8		8-8-8	8-9-8	
N	6	9	6	8	8	7	13
V/Å^3^	3724.9	5720.9	3860.0	5205.0	5409.8	4807.8	9133.3
A/Å^2^	1502.5	2152.9	1559.7	1895.4	2020.2	1807.7	3103.6
R/Å	9.62	11.09	9.73	10.75	10.89	10.47	12.97
d/g cm^−3^	1.0666	1.0417	1.0655	1.0535	1.0481	1.0658	1.0419
R_g_/Å	7.47	9.16	7.71	8.61	8.72	8.20	10.52

N—an assumed number of building units, V—volume of micelle, A—area of micelle, R—radius of same volume sphere, R_g_—radius of gyration.

**Table 2:** Temperature dependence of micellisation parameters: critical micelle concentration (c.m.c), C_m.trans_ and thermodynamic quantities of the micellisation and transformation process for aqueous surfactant solution 8-8-8 determined on the basis of calorimetric titration. The values for the micelle transformation process are shown in bold.

*T* /K	*c.m.c*/mol dm^−3^*C_m_._trans_*/mol dm^−3^	Thermodynamic Functions for 8-8-8			
		$\Delta G_{m}/\Delta G_{m.trans}$ /kJ mol^−1^	$\Delta H_{m}/\Delta H_{m.trans}$ /kJ mol^−1^	$T\Delta S_{m}/T\Delta S_{m.trans}$ /kJ mol^−1^	$\Delta S_{m}/\Delta S_{m.trans}$ /J mol^−1^ K^−1^
283.15	0.092 ± 0.09	−17.12	5.66	22.78	80.45
288.15	0.082 ± 0.08	−17.57	5.80	23.37	81.12
293.15	0.077 ± 0.007	−17.90	4.52	22.42	76.45
298.15	0.071 ± 0.007	−18.27	3.67	21.94	73.58
303.15	0.065 ± 0.006	−18.64	3.29	21.93	72.33
308.15	0.062 ± 0.006	−18.90	2.31	21.20	68.80
313.15	0.063 ± 0.006	−19.02	1.39	20.41	65.17
318.15	0.049 ± 0.005 **0.116 ± 0.008**	−19.83 **−17.40**	1.36 **−2.10**	21.19 **15.30**	66.60 **48.09**
323.15	0.032 ± 0.003 **0.088 ± 0.005**	−21.15 **−18.29**	0.47 **−2.16**	21.62 **16.14**	66.91 **49.95**
328.15	0.023 ± 0.002 **0.113 ± 0.01**	−22.22 **−17.67**	0.52 **−2.82**	22.74 **14.85**	69.29 **45.25**
333.15	0.122 ± 0.01	−17.56	−3.99	13.57	40.73
338.15	0.130 ± 0.01	−17.46	−4.10	13.36	39.52
343.15	0.145 ± 0.01	−17.24	−4.05	13.19	38.44

**Table 3:** SCF energies (E_SCF_): kinetic E_SCF-kin._ and potential E_SCF-pot._ of surfactant conformers in aqueous solutions optimized at the B3LYP/def2–TZVP.

	Conformation	E_SCF_	E_SCF-kinet_	E_SCF-pot_.
*8-6-8*	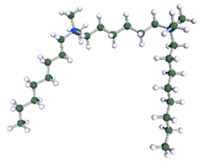	−4.95 × 10^−15^ J	−4.92 × 10^−15^ J	−9.87 × 10^−15^ J
		−2.98 × 10^6^ KJ mol^−1^	−2.96 × 10^6^ KJ mol^−1^	−5.94 × 10^−15^ KJ mol^−1^
	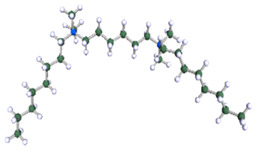	−4.95 × 10^−15^ J	−4.92 × 10^−15^ J	−9.87 × 10^−15^ J
		−2.98 × 10^6^ KJ mol^−1^	−2.96 × 10^6^ KJ mol^−1^	−5.94 × 10^−15^ KJ mol^−1^
	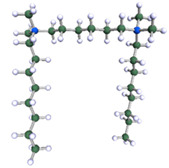	−4.95 × 10^−15^ J	−4.92 × 10^−15^ J	−9.87 × 10^−15^ J
		−2.98 × 10^6^ KJ mol^−1^	−2.96 × 10^6^ KJ mol^−1^	−5.94 × 10^−15^ KJ mol^−1^
*8-7-8*	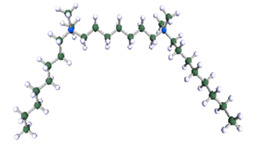	−5.12 × 10^−15^ J	*−5.09 × 10^−15^ J*	*−1.02 × 10^−14^ J*
		−3.08 × 10^6^ KJ mol^−1^	−3.07 × 10^6^ KJ mol^−1^	−6.15 × 10^6^ KJ mol^−1^
	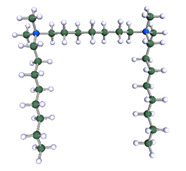	−5.12 × 10^−15^ J	−5.09 × 10^−15^ J	−1.02 × 10^−14^ J
		−3.08 × 10^6^ KJ mol^−1^	−3.07 × 10^6^ KJ mol^−1^	−6.15 × 10^6^ KJ mol^−1^
	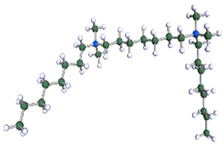	−5.12 × 10^−15^ J	*−5.09 × 10^−15^ J*	*−1.02 × 10^−14^ J*
		*−3.08 × 10^6^ KJ mol^−1^*	*−3.07 × 10^6^ KJ mol^−1^*	*−6.15 × 10^6^ KJ mol^−1^*
*8-8-8*	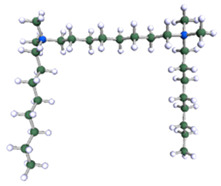	−5.29 × 10^−15^ J	−5.26 × 10^−15^ J	−1.05 × 10^−14^ J
		−3.18 × 10^6^ KJ mol^−1^	−3.17 × 10^6^ KJ mol^−1^	−6.35 × 10^6^ KJ mol^−1^
	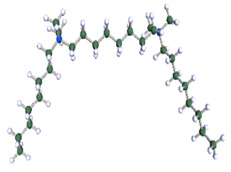	−5.29 × 10^−15^ J	−5.26 × 10^−15^ J	−1.05 × 10^−14^ J
		−3.18 × 10^6^ KJ mol^−1^	−3.17 × 10^6^ KJ mol^−1^	−6.35 × 10^6^ KJ mol^−1^
	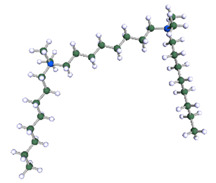	−5.29 × 10^−15^ J	−5.26 × 10^−15^ J	−1.05 × 10^−14^ J
		−3.18 × 10^6^ KJ mol^−1^	−3.17 × 10^6^ KJ mol^−1^	−6.35 × 10^6^ KJ mol^−1^
*8-9-8*	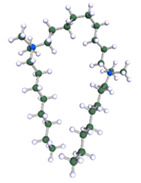	−5.46 × 10^−15^ J	−5.43 × 10^−15^ J	−1.09 × 10^−14^ J
		−3.29 × 10^6^ KJ mol^−1^	−3.27 × 10^6^ KJ mol^−1^	−6.56 × 10^6^ KJ mol^−1^
	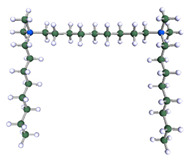	−5.46 × 10^−15^ J	−5.43 × 10^−15^ J	−1.09 × 10^−14^ J
		−3.29 × 10^6^ KJ mol^−1^	−3.27 × 10^6^ KJ mol^−1^	−6.56 × 10^6^ KJ mol^−1^

## Supplementary Materials

Supplementary Materials can be found at https://www.mdpi.com/1422-0067/21/16/5828/s1.
