# Supplementary material for: Can the Isothermal Calorimetric Curve Shapes Suggest the Structural Changes in Micellar Aggregates?
Source: Int J Mol Sci. 2020 Aug 13;21(16):5828. doi: 10.3390/ijms21165828 (PMC7461569; doi:10.3390/ijms21165828)
Supplement: Supplementary file 1 [file ijms-21-05828-s001.pdf]

Figure S1. ^{1}H-NMR spectra for heksylene-1,6-bis(dimethyloctylammonium) bromides.

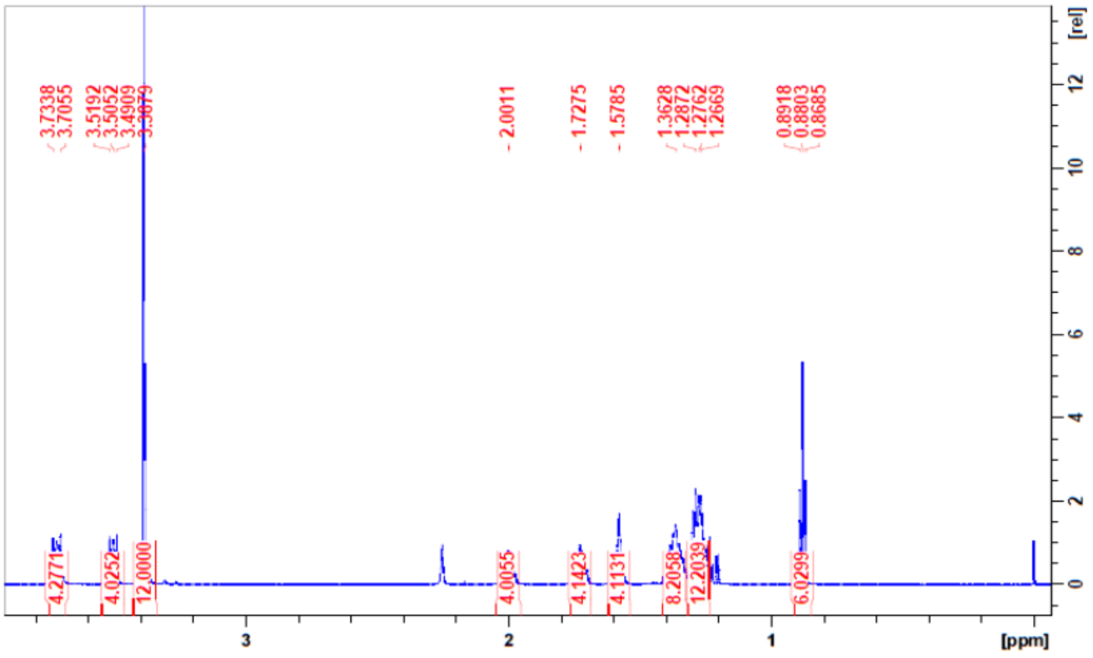

^{1}H-NMR (600 MHz, $CDCl_3$): 0.88 (t, 6H, *J* = 6.9, 2xCH_3), 1.22-1.26 (m, 12H, 6xCH_2), 1.26-1.31 (m, 8H, 4xCH_2), 1.56-1.61 (m, 4H, 2xCH_2), 1.64-1.68 (m, 4H, 2xCH_2), 1.80-2.05 (m, 4H, 2xCH_2), 3.39 (s, 12H, 4xCH_3N), 3.45-3.55 (m, 4H, 2xCH_2N), 3.65-3.75 (m, 4H, 2xCH_2N).

Figure S2. ^{13}C-NMR spectra for heksylene-1,6-bis(dimethyloctylammonium) bromides.

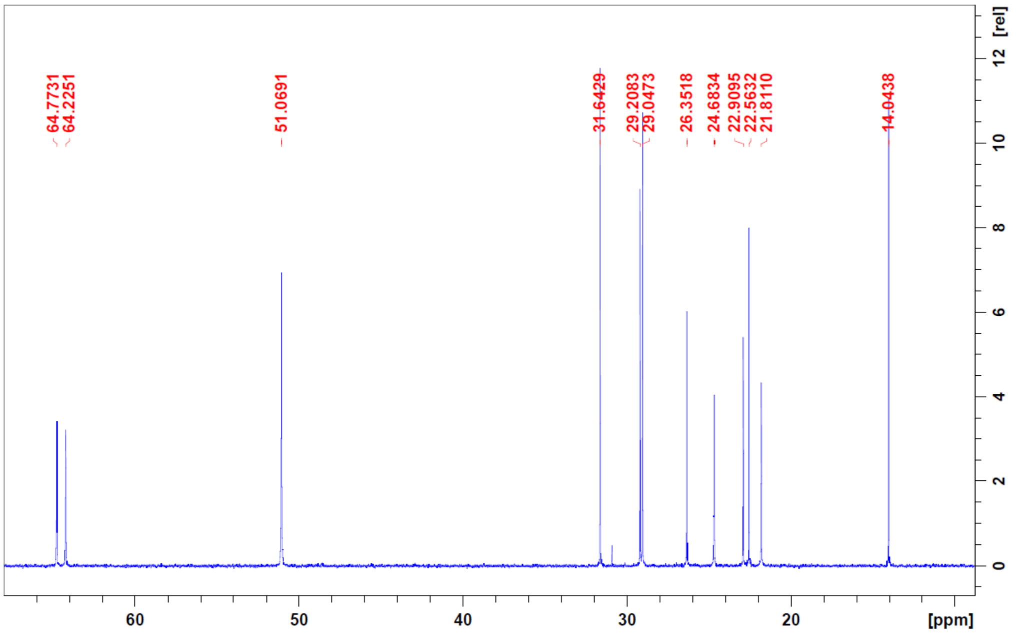

^{13}C-NMR (150 MHz, $CDCl_3$): 14.04 (2xCH_3), 21.81, 22.65, 22.90, 24.63, 26.35, 29.05, 29.21, 31.64 (16xCH_2), 51.06 (4xCH_3N), 64.23 (2xCH_2N), 64.77 (2xCH_2N).

Figure S3. ^{1}H-NMR spectra for heptylene-1,7-bis(dimethyloctylammonium) bromides.

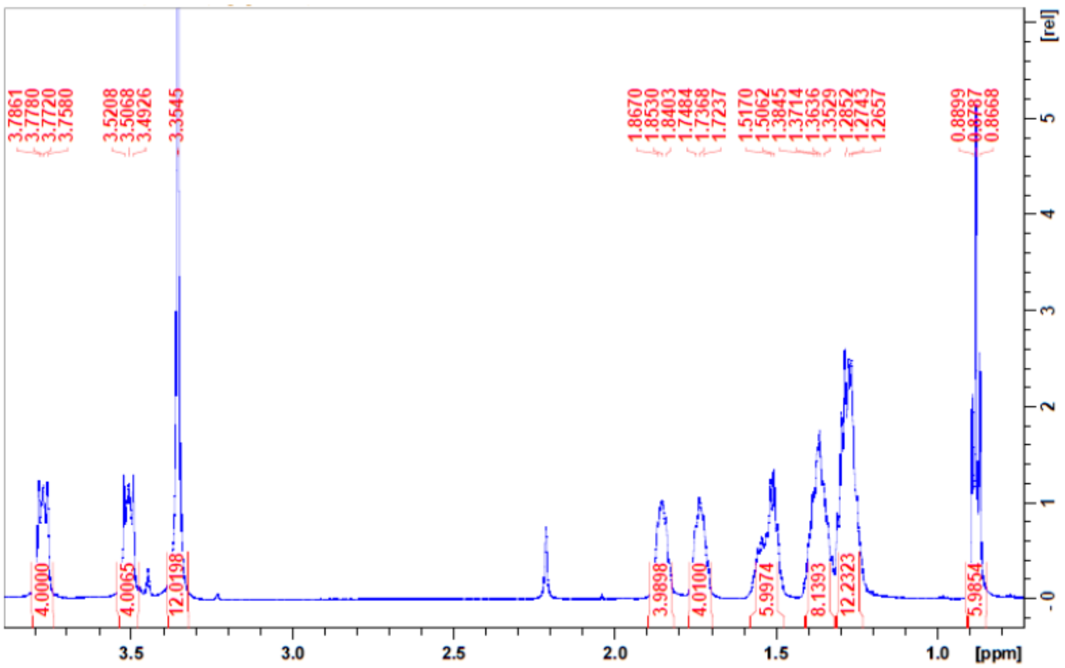

^{1}H-NMR (600 MHz, $CDCl_3$): 0.88 (t, 6H, J = 6.7, $2xCH_3$), 1.22-1.31 (m, 12H, $6xCH_2$), 1.31-1.41 (m, 8H, $4xCH_2$), 1.47-1.58 (m, 6H, $3xCH_2$), 1.70-1.77 (m, 4H, $2xCH_2$), 1.82-1.89 (m, 4H, $2xCH_2$), 3.35 (s, 12H, $4xCH_3N$), 3.48-3.54 (m, 4H, $2xCH_2N$), 3.74-3.81 (m, 4H, $2xCH_2N$).

Figure S4. ^{13}C-NMR spectra for heptylene-1,7-bis(dimethyloctylammonium) bromides.

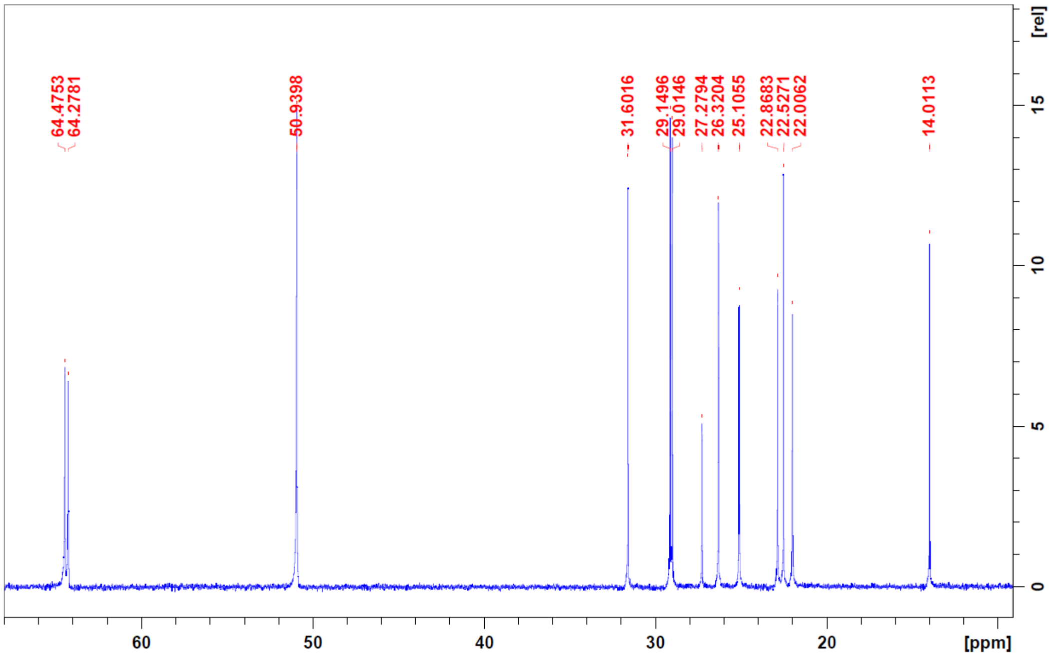

^{13}C-NMR (150 MHz, $CDCl_3$): 14.08 ($2xCH_3$), 22.03, 22.60, 22.93, 25.11, 26.39, 27.23, 29.08, 29.22, 31.67 ($17xCH_2$), 50.98 ($4xCH_3N$), 64.39 ($2xCH_2N$), 64.57 ($2xCH_2N$).

Figure S5. ^{1}H-NMR spectra for oktylene-1,8-bis(dimethyloctylammonium) bromides

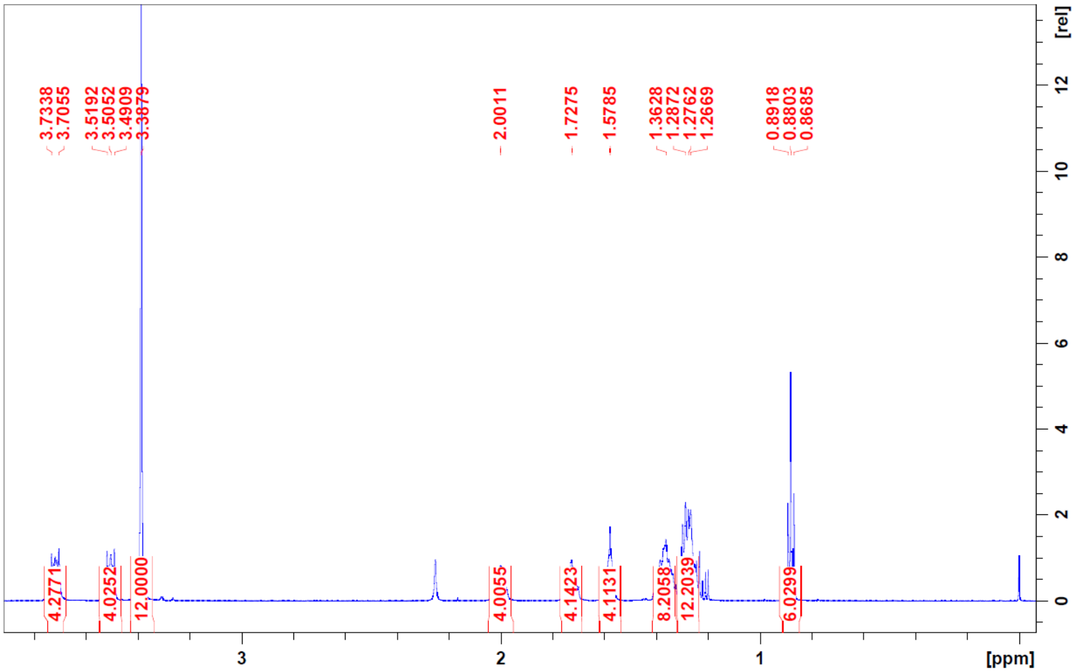

^{1}H-NMR (600 MHz, $CDCl_3$): 0.88 (t, 6H, J = 7.0, 2xCH_3), 1.22-1.32 (m, 12H, 6xCH_2), 1.32-1.42 (m, 8H, 4xCH_2), 1.43-1.50 (m, 8H, 4xCH_2), 1.69-1.75 (m, 4H, 2xCH_2), 1.83-1.89 (m, 4H, 2xCH_2), 3.37 (s, 12H, 4xCH_3N), 3.50-3.56 (m, 4H, 2xCH_2N), 3.66-3.74 (m, 4H, 2xCH_2N).

Figure S6. ^{13}C-NMR spectra for oktylene-1,8-bis(dimethyloctylammonium) bromides

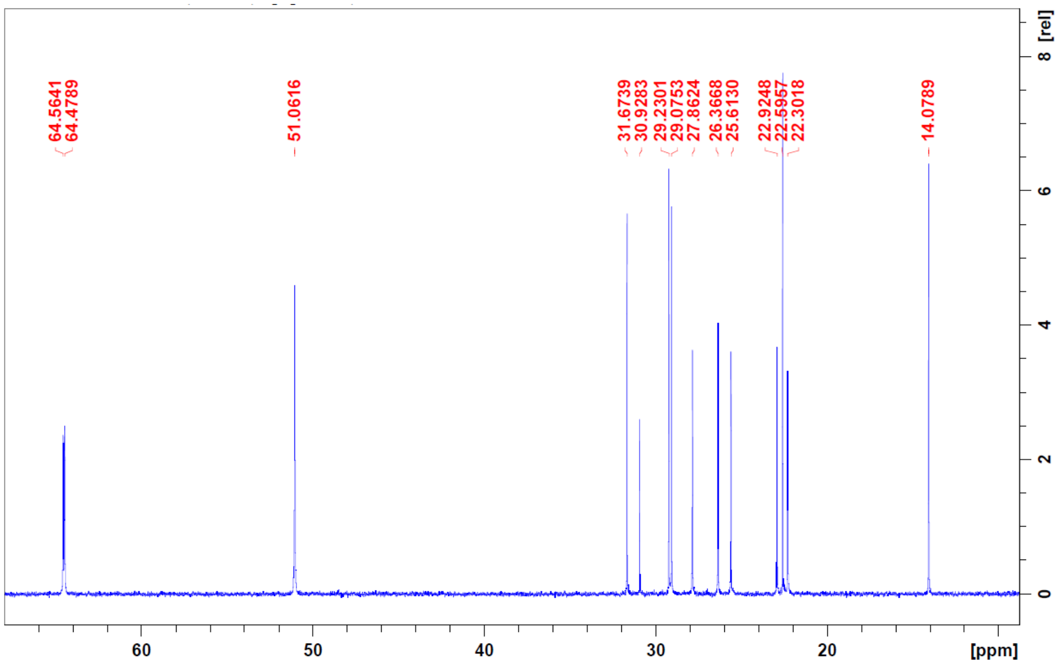

^{13}C-NMR (150 MHz, $CDCl_3$): 14.09 (2xCH_3), 22.32, 22.62, 22.95, 25.62, 26.39, 27.85, 29.09, 29.25, 31.70 (18xCH_2), 51.06 (4xCH_3N), 64.52 (2xCH_2N), 64.62 (2xCH_2N).

Figure S7. ^{1}H-NMR spectra for nonylene-1,9-bis(dimethyloctylammonium) bromides

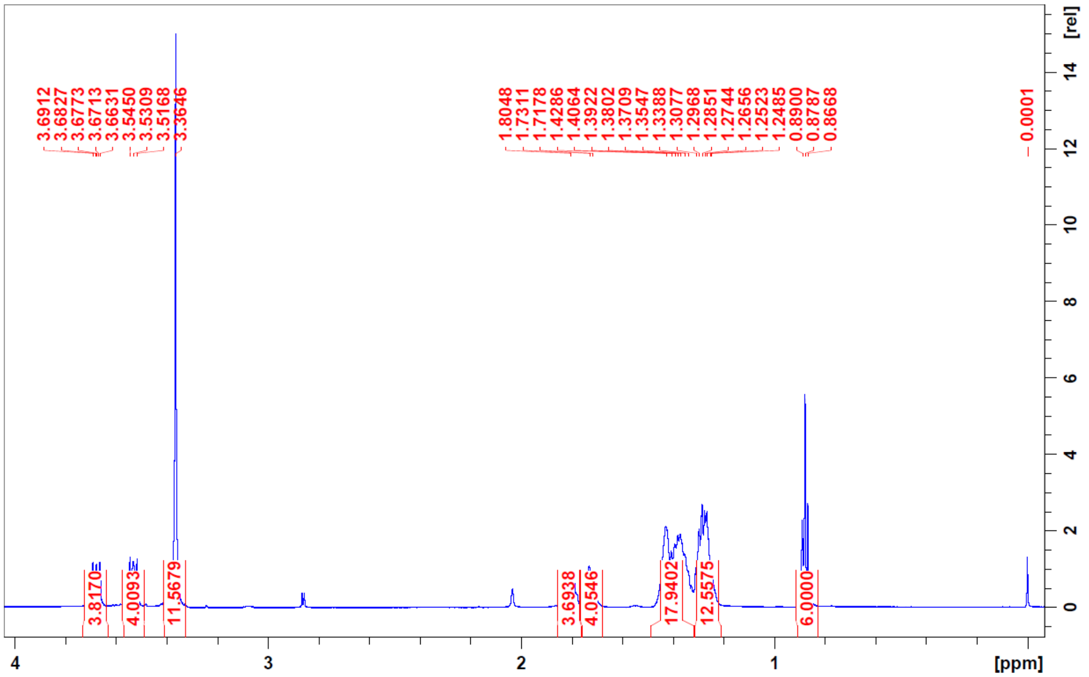

^{1}H-NMR (600 MHz, $CDCl_3$): 0.88 (t, 6H, J = 7.0, 2xCH_3), 1.21-1.32 (m, 12H, 6xCH_2), 1.32-1.48 (m, 18H, 4xCH_2), 1.68-1.75 (m, 4H, 2xCH_2), 1.76-1.85 (m, 4H, 2xCH_2), 3.36 (s, 12H, 4xCH_3N), 3.50-3.57 (m, 4H, 2xCH_2N), 3.64-3.72 (m, 4H, 2xCH_2N)

Figure S8. ^{13}C-NMR spectra for nonylene-1,9-bis(dimethyloctylammonium) bromides

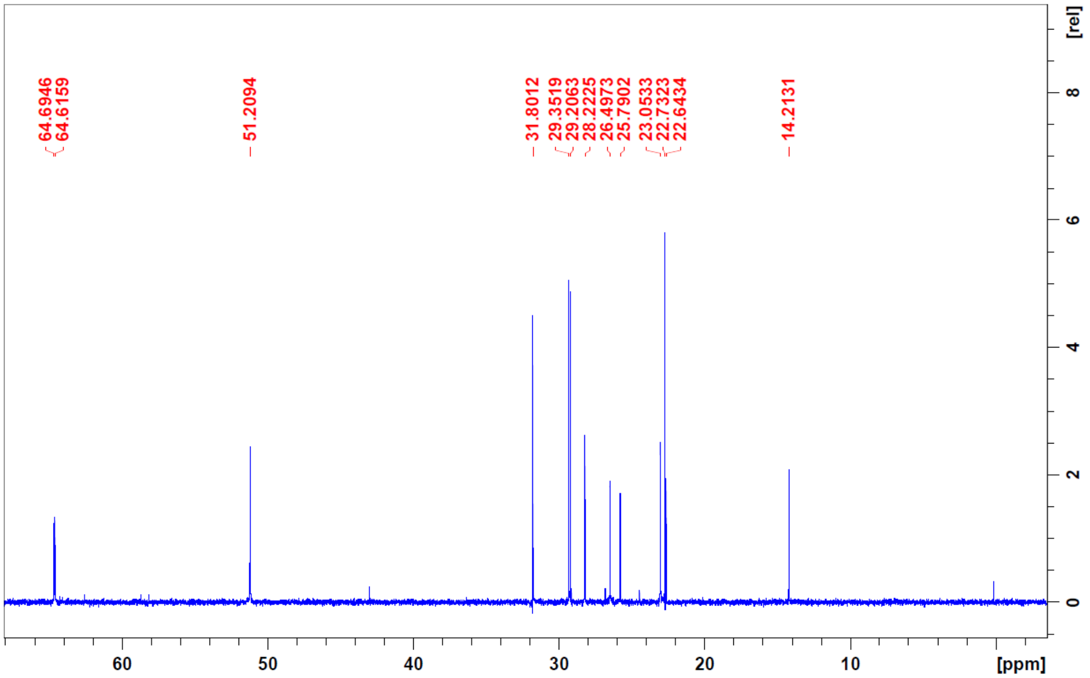

^{13}C-NMR (150 MHz, $CDCl_3$): 14.21 (2xCH_3), 22.64, 22.73, 223.05, 25.79, 26.50, 28.22, 29.21, 29.35, 31.80 (18xCH_2), 51.21 (4xCH_3N), 64.62 (2xCH_2N), 64.69 (2xCH_2N).

Figure S9. ^{1}H-NMR spectra for tretadecylene-1,14-bis(dimethyloctylammonium) bromides

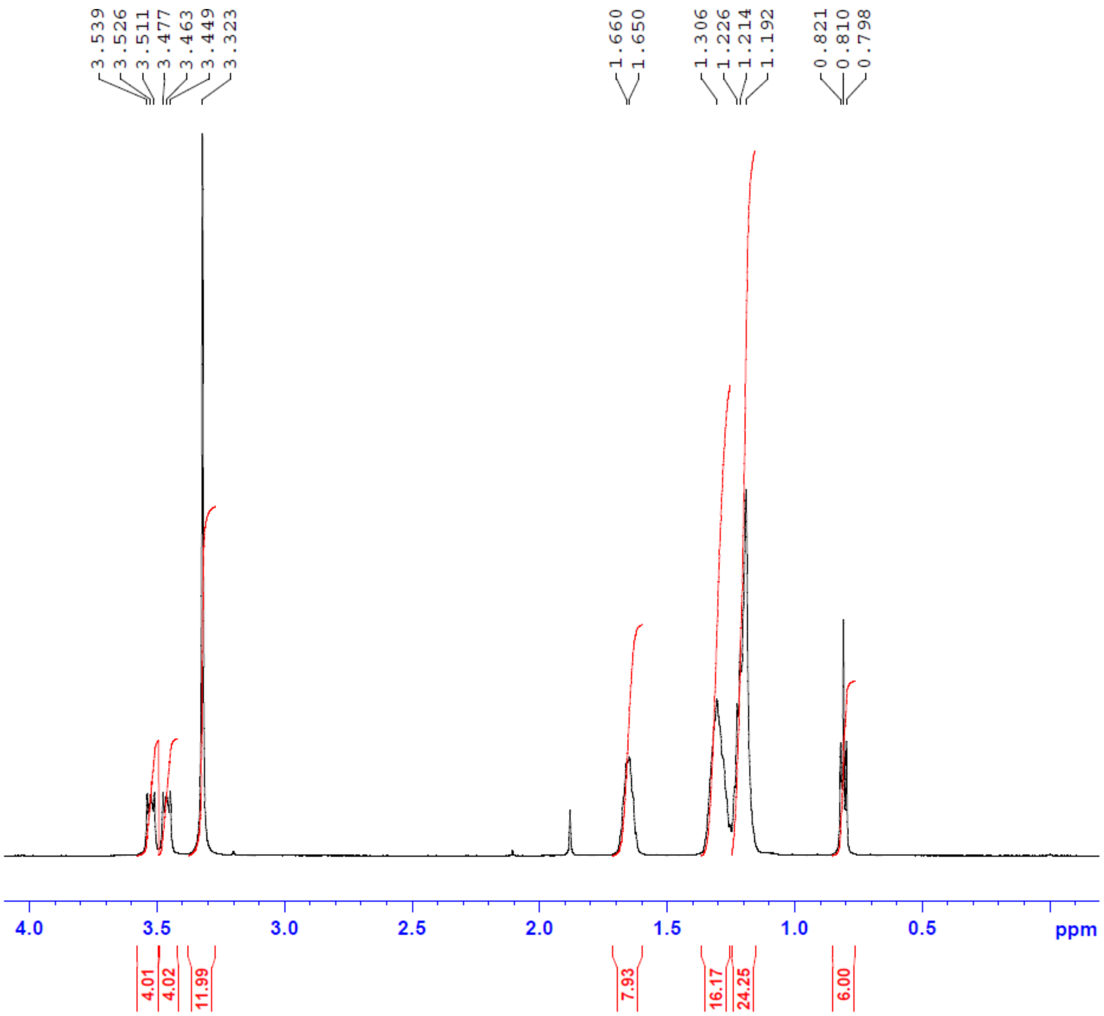

^{1}H-NMR (600 MHz, $CDCl_3$): 0.81 (t, 6H, *J* = 6.7, 2xC**H**$_3$), 1.14-1.25 (m, 24H, 12xC**H**$_2$), 1.25-1.37 (m, 16H, 8xC**H**$_2$), 1.59-1.71 (m, 8H, 4xC**H**$_2$), 3.32 (s, 12H, 4xC**H**$_3$N), 3.42-3.49 (m, 4H, 2xC**H**$_2$N), 3.49-3.57 (m, 4H, 2xC**H**$_2$N).

Figure S10. ^{13}C-NMR spectra for tretadecylene-1,14-bis(dimethyloctylammonium) bromide

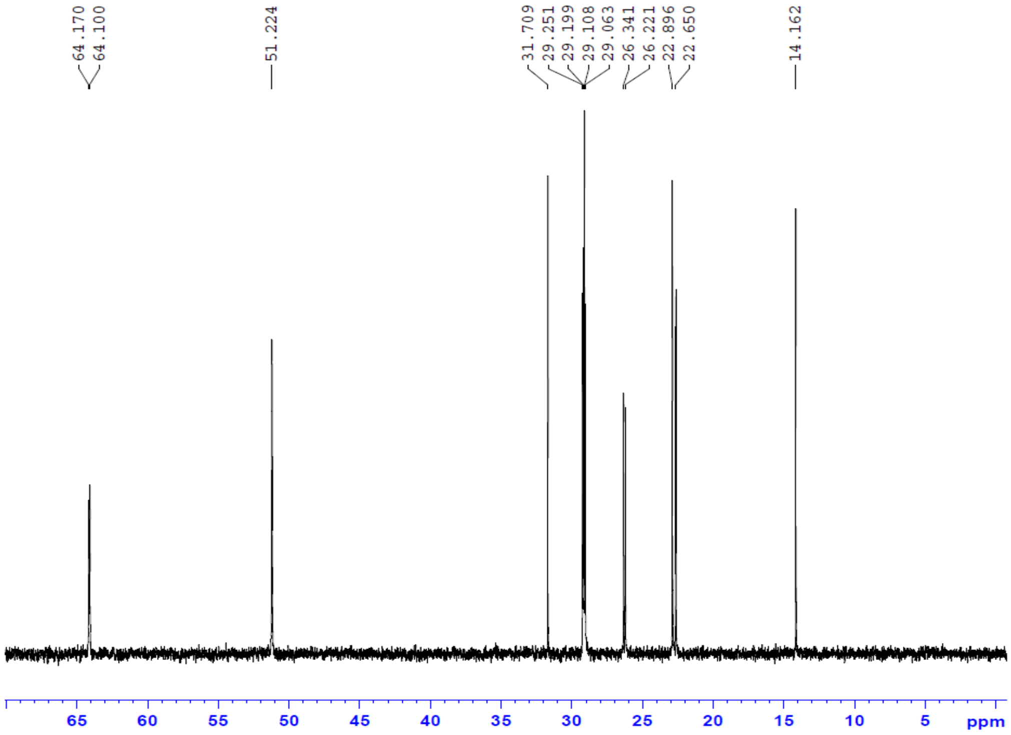

^{13}C-NMR (150 MHz, $CDCl_3$): 14.16 (2x**C**H_3), 22.65, 22.89, 26.22, 26.34, 29.06, 29.11, 29.20, 29.25, 31.71 (24x**C**H_2), 51.22 (4x**C**H_3N), 64.10 (2x**C**H_2N), 64.17 (2x**C**H_2N).

Figure S11. Temperature dependence of counterion binding to micelles, β, determined for aqueous solutions of heksylene-1,6 bis(dimethyloctylammonium) bromides by the slope ratio method conductometrically.

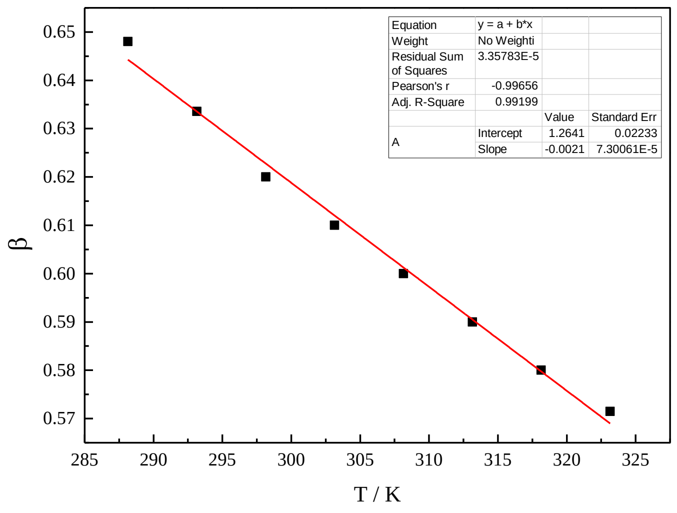

Figure S12. Temperature dependence of counterion binding to micelles, β, determined for aqueous solutions of heptylene -1,7 bis(dimethyloctylammonium) bromides by the slope ratio method conductometrically.

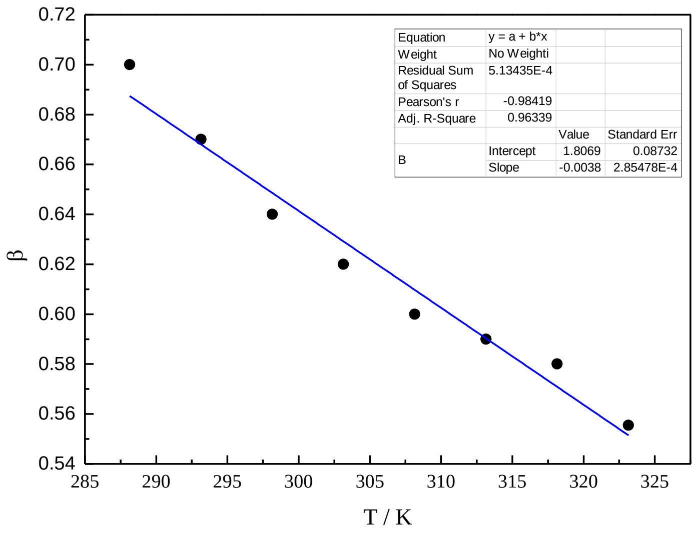

Figure S13. Temperature dependence of counterion binding to micelles, β, determined for aqueous solutions of oktylene -1,8 bis(dimethyloctylammonium) bromides by the slope ratio method conductometrically.

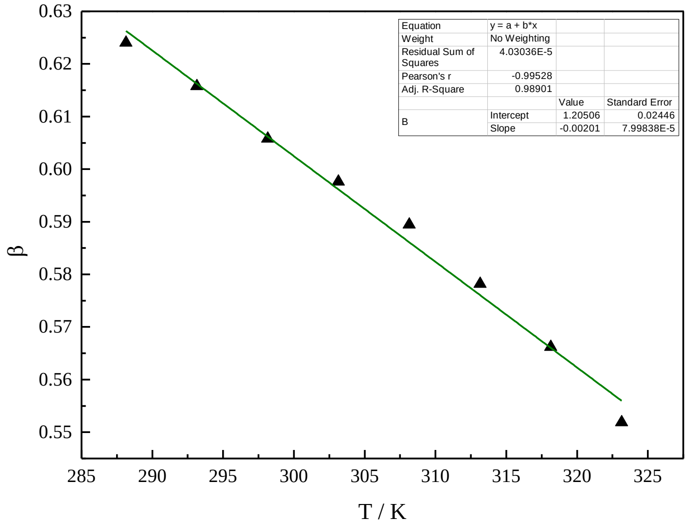

Figure S14. Temperature dependence of counterion binding to micelles, β, determined for aqueous solutions of nonylene -1,9 bis(dimethyloctylammonium) bromides by the slope ratio method conductometrically.

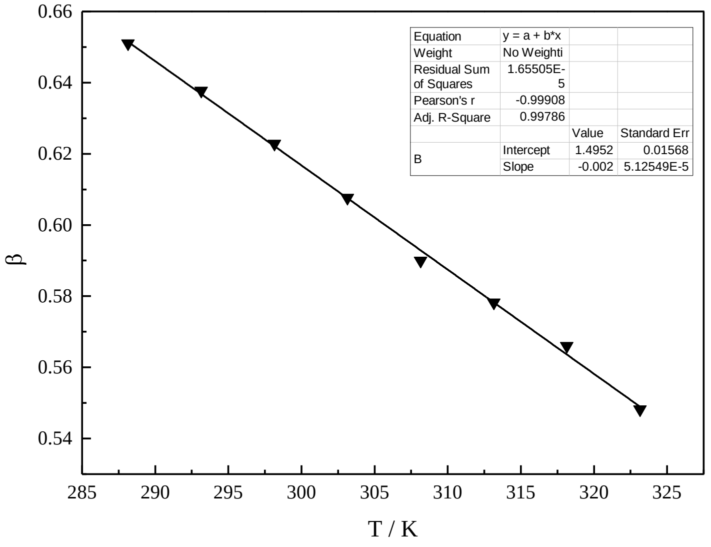

Figure S15. Temperature dependence of counterion binding to micelles, β, determined for aqueous solutions of tetradecylene -1,14 bis(dimethyloctylammonium) bromides by the slope ratio method conductometrically.

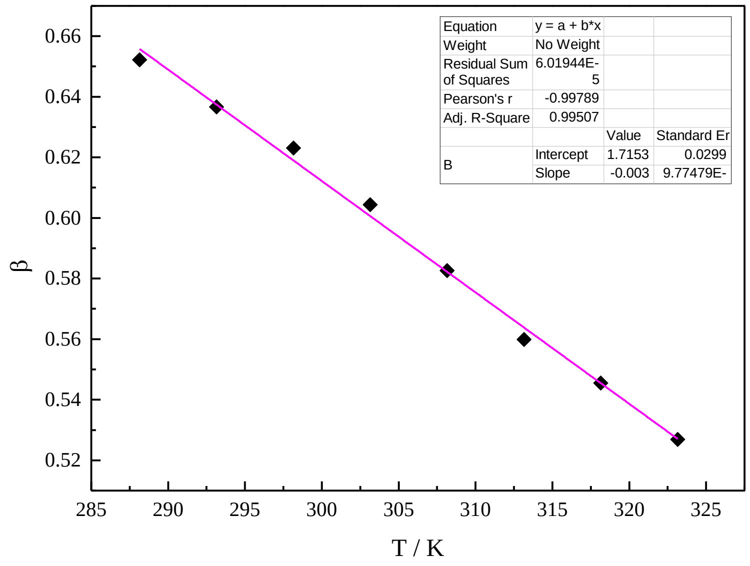

Figure S16. Calorimetric titration curve from additions 8-6-8 surfactant to water for temperatures: ○ *– 283.15 K, ∇ - 288.15 K, • - 293.15 K, ◊ - 298.15K, □ – 303.15K, Δ - 308.15K, Δ - 313.15K, • - 318.15K, ♦ - 323.15 K, ∇ - 328.15K, ♦ - 333.15 K, P – 338.15K, ∇ - 343.15 K*

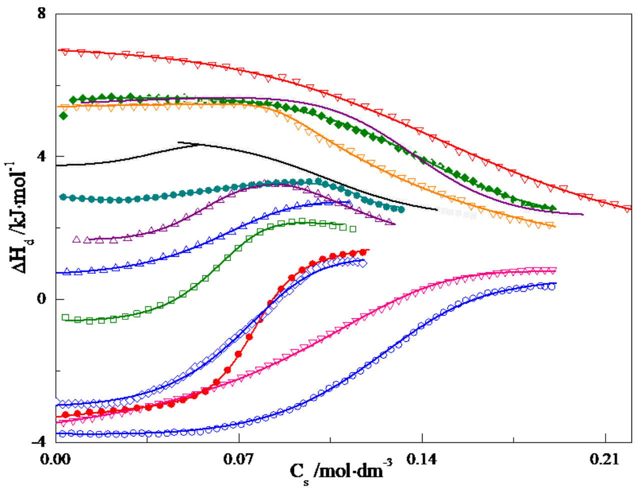

Figure S17. Calorimetric titration curve from additions 8-7-8 surfactant to water for temperatures: ○ *– 283.15 K, ∇ - 288.15 K, ● - 293.15 K, ◊ - 298.15K, □ – 303.15K, Δ - 308.15K, Δ - 313.15K, ● - 318.15K, ♦ - 323.15 K, ∇ - 328.15K, ♦ - 333.15 K, P – 338.15K, ∇ - 343.15 K*

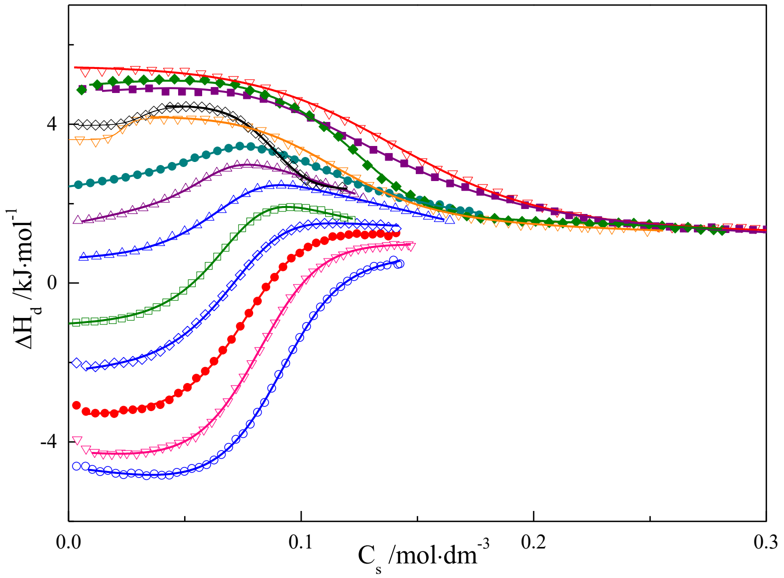

Figure S18. Calorimetric titration curve from additions 8-9-8 surfactant to water for temperatures: *∇ - 288.15 K, ● - 293.15 K, ◊ - 298.15K, □ −303.15K, Δ - 308.15K, Δ - 313.15K, ●-318.15K, ♦ - 323.15 K*

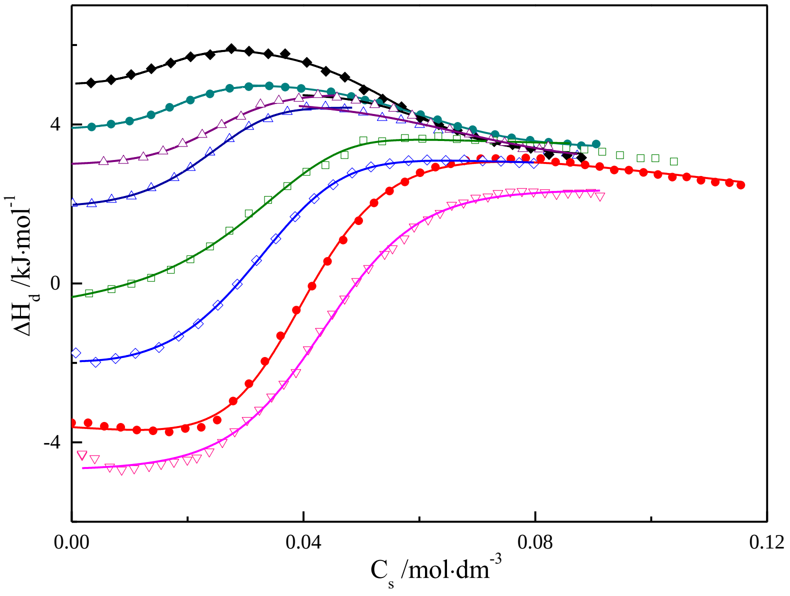

Figure S19. Pair distance distribution functions derived from SAXS data by indirect Fourier transform for 8-7-8 surfactant system at concentrations: a) 0.150 mol dm^{-3}, b) 0.214 mol dm^{-3}, c) 0.331 mol dm^{-3}, d) 0.342 mol dm^{-3}, e) 0.352 mol dm^{-3} at 293.15 K, 298.15 K, 303.15 K, 308.15 K, 313.15 K, 318.15 K .

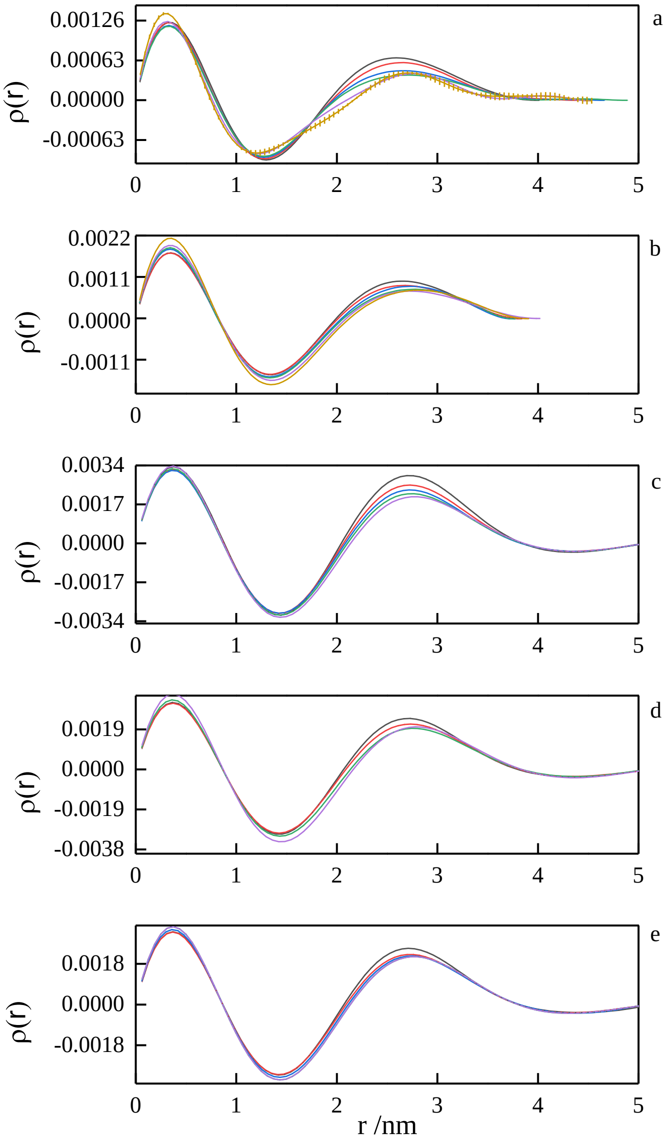

Figure S20. Pair distance distribution functions derived from SAXS data by indirect Fourier transform for 8-9-8 surfactant system at concentrations a) 0.40 mol dm^{-3},) b) 0.107 mol dm^{-3} at 293.15 K, 298.15 K, 303.15 K.

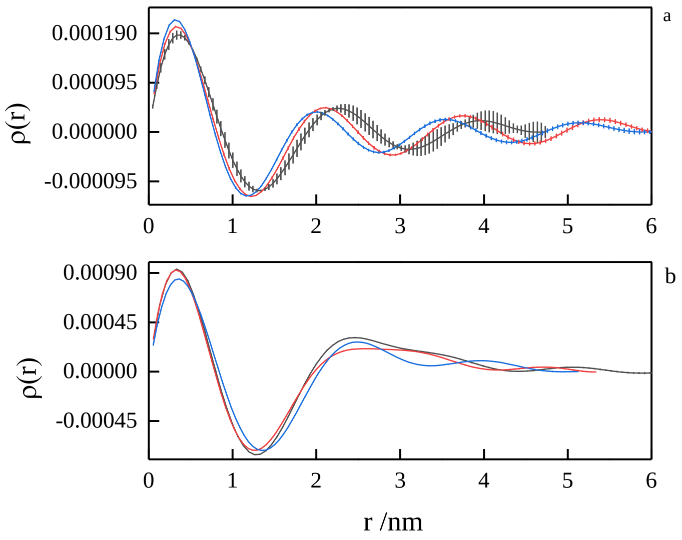

Table S1. Temperature dependence of micellisation parameters: c.m.c, $C_{m.trans}$ and thermodynamic quantities of the micellisation and transformation process for aqueous surfactant solution 8-6-8 determined on the basis of calorimetric titration. The values for the micelle transformation process are shown in bold.

T /K	$c.m.c$ /mol · dm^{-3} $C_{m\cdot trans}$ /mol · dm^{-3}	Thermodynamic Functions for 8-6-8			
		ΔG_m / $\boldsymbol{\Delta G_{m.trans}}$ /kJ · mol^{-1}	ΔH_m / $\boldsymbol{\Delta H_{m.trans}}$ /kJ · mol^{-1}	$T\Delta S_m$ / $\boldsymbol{T\Delta S_{m.trans}}$ /kJ · mol^{-1}	ΔS_m / $\boldsymbol{\Delta S_{m.trans}}$ /J · mol^{-1} · K^{-1}
283.15	0.126 ±0.01	-16.57	4.11	20.68	73.04
288.15	0.106 ±0.01	-17.17	3.70	20.86	72.41
293.15	0.077 ±0.007	-18.12	3.60	21.72	74.10
298.15	0.070 ±0.007	-18.59	3.55	22.14	74.25
303.15	0.063 ±0.005	-19.00	3.03	22.04	72.25
308.15	0.065 ±0.006	-19.06	2.30	21.35	69.28
313.15	0.057 ±0.006	-19.55	1.68	21.23	67.80
	**0.107 ±0.01**	**-17.76**	**-1.13**	**16.63**	**53.12**
318.15	0.053 ±0.005	-19.87	0.51	20.38	64.07
	**0.114 ±0.01**	**-17.69**	**-0.49**	**17.20**	**54.06**
323.15	0.037 ±0.005	-20.94	0.58	21.42	66.29
	**0.113 ±0.01**	**-17.89**	**-2.57**	**15.28**	**47.28**
328.15	0.116 ±0.01	-17.83	-3.90	13.93	42.44
333.15	0.138 ±0.01	-17.42	-3.56	13.86	41.60
338.15	0.136 ±0.01	-17.54	-4.07	13.46	39.81
343.15	0.147 ±0.01	-17.39	-4.95	12.43	36.23

Table S2. Temperature dependence of micellisation parameters: c.m.c, $C_{m.trans}$ and thermodynamic quantities of the micellisation and transformation process for aqueous surfactant solution 8-7-8 determined on the basis of calorimetric titration. The values for the micelle transformation process are shown in bold.

		**Thermodynamic Functions for 8-7-8**			
T /K	$c.m.c$ /mol · dm^{-3} $C_{m\cdot trans}$ /mol · dm^{-3}	ΔG_m / $\boldsymbol{\Delta G_{m.trans}}$ /kJ · mol^{-1}	ΔH_m / $\boldsymbol{\Delta H_{m.trans}}$ /kJ · mol^{-1}	$T\Delta S_m$ / $\boldsymbol{T\Delta S_{m.trans}}$ /kJ · mol^{-1}	ΔS_m / $\boldsymbol{\Delta S_{m.trans}}$ /J · mol^{-1} · K^{-1}
283.15	0.130±0.01	-17.19	3.58	20.77	73.35
288.15	0.116±0.01	-17.53	3.52	21.05	73.05
293.15	0.096±0.01	-18.08	3.28	21.36	72.86
298.15	0.090±0.01	-18.27	2.88	21.15	70.43
303.15	0.081±0.008	-18.55	2.41	20.96	69.14
308.15	0.079±0.008	-18.61	1.34	19.95	64.74
313.15	0.074±0.007	-18.77	0.73	19.50	62.27
318.15	0.055±0.005	-19.57	0.43	20.00	62.86
	**0.130±0.01**	**-17.14**	**-0.48**	**16.66**	**52.37**
323.15	0.049±0.005	-19.84	0.36	20.20	62.51
	**0.139±0.01**	**-16.90**	**-1.14**	**15.76**	**48.77**
328.15	0.136±0.01	-16.91	-1.90	15.01	45.74
333.15	0.142±0.01	-16.72	-1.80	14.92	44.78
338.15	0.147±0.01	-16.55	-1.83	14.72	43.53
343.15	0.160±0.01	-16.23	-1.67	14.56	42.43

Table S3. Temperature dependence of micellisation parameters: c.m.c, $C_{m.trans}$ and thermodynamic quantities of the micellisation and transformation process for aqueous surfactant solution 8-9-8 determined on the basis of calorimetric titration. The values for the micelle transformation process are shown in bold.

		**Thermodynamic Functions for 8-9-8**			
T /K	$c.m.c$ /mol · dm^{-3} $C_{m\cdot trans}$ /mol · dm^{-3}	ΔG_m / $\boldsymbol{\Delta G_{m.trans}}$ /kJ · mol^{-1}	ΔH_m / $\boldsymbol{\Delta H_{m.trans}}$ /kJ · mol^{-1}	$T\Delta S_m$ / $\boldsymbol{T\Delta S_{m.trans}}$ /kJ · mol^{-1}	ΔS_m / $\boldsymbol{\Delta S_{m.trans}}$ /J · mol^{-1} · K^{-1}
288.15	0.043±0.004	-19.98	7.15	27.13	95.81
293.15	0.039±0.004	-20.09	7.89	27.98	95.44
298.15	0.034±0.003	-20.56	6.06	26.62	89.28
303.15	0.032±0.003	-20.80	3.92	24.72	81.54
308.15	0.025±0.002	-21.57	2.67	24.24	78.68
	**0.065±0.006**	**-18.88**	**-1.46**	**17.42**	**56.52**
313.15	0.024±0.002	-21.69	1.80	23.49	75.02
	**0.062±0.006**	**-19.06**	**-1.49**	**17.57**	**56.12**
318.15	0.018±0.001	-22.59	1.14	23.73	74.59
	**0.060±0.006**	**-19.21**	**-2.02**	**17.19**	**54.03**
323.15	0.015±0.001	-23.08	0.93	24.02	74.32
	**0.058±0.005**	**-19.49**	**-2.80**	**16.69**	**51.65**

Table S4. Temperature dependence of micellisation parameters: c.m.c, $C_{m.trans}$ and thermodynamic quantities of the micellisation and transformation process for aqueous surfactant solution 8-14-8 determined on the basis of calorimetric titration. The values for the micelle transformation process are shown in bold.

T /K	$c.m.c$ /mol · dm^{-3} $C_{m.trans}$ /mol · dm^{-3}	**Thermodynamic Functions for 8-14-8** ΔG_m / $\boldsymbol{\Delta G_{m.trans}}$ /kJ · mol^{-1}	ΔH_m / $\boldsymbol{\Delta H_{m.trans}}$ /kJ · mol^{-1}	$T\Delta S_m$ / $\boldsymbol{T\Delta S_{m.trans}}$ /kJ · mol^{-1}	ΔS_m / $\boldsymbol{\Delta S_{m.trans}}$ /J · mol^{-1} · K^{-1}
283.15	0.022 ±0.001	-21.58	10.46	32.04	113.2
288.15	0.021 ±0.001	-21.83	8.92	30.75	106.7
293.15	0.020 ±0.001	-22.04	6.77	28.81	98.27
298.15	0.017 ±0.001	-22.36	5.72	28.08	94.19
303.15	0.017 ±0.001	-22.44	3.61	26.05	85.92
	**0.044 ±0.001**	**-19.73**	**-2.33**	**17.40**	**57.40**
308.15	0.011 ±0.001	-23.63	3.39	27.02	87.68
	**0.041 ±0.001**	**-19.98**	**-2.57**	**17.41**	**56.51**
313.15	0.007 ±0.001	-24.86	2.82	27.67	88.37
	**0.028 ±0.001**	**-21.02**	**-4.85**	**16.17**	**51.62**
318.15	0.026 ±0.002	-21.18	-6.02	15.16	47.66
323.15	0.027 ±0.003	-21.04	-7.22	13.82	42.76
328.15	0.028 ±0.003	-20.88	-8.70	12.18	37.12
333.15	0.028 ±0.003	-20.81	-10.56	10.24	30.76
338.15	0.029 ±0.003	-20.63	-10.57	10.06	29.76
343.15	0.030 ±0.003	-20.45	-12.08	8.37	24.40
